# Fin whale movements in the Gulf of California, Mexico, from satellite telemetry

**DOI:** 10.1371/journal.pone.0209324

**Published:** 2019-01-10

**Authors:** M. Esther Jiménez López, Daniel M. Palacios, Armando Jaramillo Legorreta, Jorge Urbán R., Bruce R. Mate

**Affiliations:** 1 Programa de Investigación de Mamíferos Marinos. Departamento Académico de Ciencias Marinas y Costeras, Universidad Autónoma de Baja California Sur, La Paz, Baja California Sur, México, Mezquitito, La Paz, México; 2 Marine Mammal Institute and Department of Fisheries and Wildlife, Oregon State University, Hatfield Marine Science Center, Newport, Oregon, United States of America; 3 National Institute of Ecology. Marine Mammals Program Camper #6. Ensenada, B.C. México; Texas A&M University, UNITED STATES

## Abstract

Fin whales (*Balaenoptera physalus*) have a global distribution, but the population inhabiting the Gulf of California (GoC) is thought to be geographically and genetically isolated. However, their distribution and movements are poorly known. The goal of this study was to describe fin whale movements for the first time from 11 Argos satellite tags deployed in the southwest GoC in March 2001. A Bayesian Switching State-Space Model was applied to obtain improved locations and to characterize movement behavior as either “area-restricted searching” (indicative of patch residence, ARS) or “transiting” (indicative of moving between patches). Model performance was assessed with convergence diagnostics and by examining the distribution of the deviance and the behavioral parameters from Markov Chain Monte Carlo models. ARS was the predominant mode behavior 83% of the time during both the cool (December-May) and warm seasons (June-November), with slower travel speeds (mean = 0.84 km/h) than during transiting mode (mean = 3.38 km/h). We suggest ARS mode indicates either foraging activities (year around) or reproductive activities during the winter (cool season). We tagged during the cool season, when the whales were located in the Loreto-La Paz Corridor in the southwestern GoC, close to the shoreline. As the season progressed, individuals moved northward to the Midriff Islands and the upper gulf for the warm season, much farther from shore. One tag lasted long enough to document a whale’s return to Loreto the following cool season. One whale that was originally of undetermined sex, was tagged in the Bay of La Paz and was photographed 10 years later with a calf in the nearby San Jose Channel, suggesting seasonal site fidelity. The tagged whales moved along the western GoC to the upper gulf seasonally and did not transit to the eastern GoC south of the Midriff Islands. No tagged whales left the GoC, providing supporting evidence that these fin whales are a resident population.

## Introduction

Population ecology has traditionally focused on understanding temporal fluctuations of animal abundance, but how animals move over time is fundamental for understanding population processes, and it is still relatively poorly understood[1]. Movement is an essential component in the life history and habitat use of individuals [2]. Movement is defined as a change in the spatial location of an individual in time, driven by processes that act across multiple spatial and temporal scales, as a strategy for locating suitable breeding or feeding habitats, moving toward or away from conspecifics, or just to relocate [3,4]. Understanding animal movement is also important for wildlife conservation planners, who are interested in maintaining the connectivity between designated nature reserves across large geographic areas [5].

The development of Argos Platform Terminal Transmitter (PTT) technology for satellite telemetry [6,7] has played an important role in movement and migration studies for many species around the world, especially thanks to advances in spatio-temporal data collection methods [5]. While the deployment of tracking devices can impact the welfare of equipped animals, and both tags and deployment efforts can be expensive [8–10], the benefits often outweigh these costs. Through the use of telemetry, it is currently possible to track and record data about an animal’s survival, reproduction, behavior, and physiology [8]. Recent tags characterize the horizontal and vertical movements of individuals while recording their physiological state, as they travel across entire continents or to the most remote regions of the world´s oceans [11–14]. Despite the iconic status of baleen whales in conservation, our knowledge about their movements is still scarce for many species due to the remoteness or seasonal inaccessibility of their habitats. In some cases their abundance is low, further complicating data collection [15–17].

Understanding the causes of movement has been identified as one of the key challenges in the ecology of marine megafauna [18]. Fortunately, the development of electronic tags and statistical methods to analyze animal movement has improved baleen whale ecology research substantially [19–22]. Among these, Bayesian switching state-space modeling (SSM) has been used to analyze animal movement from satellite telemetry [23–26] and extended to make inferences about behavioral modalities, enabling a better understanding of the interaction between an animal’s behavior and its environment [24,26,27].

The distribution and population structure of the globally distributed fin whales (*Balaenoptera physalus*) appears to be more complex than previously thought. Their migration patterns do not conform to seasonal movement from summer feeding grounds to winter breeding grounds traditionally posited for baleen whales [28,29]. Some fin whale populations stray from the traditional whale migration framework by occurring in isolation, like those from the Mediterranean Sea [29–31] and the Gulf of California (GoC) [32,33].

Genetic and acoustic evidence suggests that fin whales from the GoC constitute a unique and apparently isolated population in the Eastern North Pacific Ocean [34,35]. Existing evidence indicates an increased presence in winter and spring in some areas of the GoC, possibly related to prey availability, and a subsequent decline during the summer when their primary prey is least abundant, suggesting some seasonal movements for this population within the GoC [33,36]. Additionally, occasional temporal and spatial overlap in song types with other populations suggests an exchange from the adjacent Eastern North Pacific [36–38]. Available data neither support nor refute the hypothesis that fin whales are residents of the GoC. Sighting data from the southern GoC and the Pacific coast of Baja California give no indication that fin whales migrate between the GoC and the Pacific Ocean [36]. This is the main reason previous researchers have recommended satellite tagging in addition to genetic and photo-identification techniques as valuable tools for examining this hypothesis and for better describe their movements [39].

Information about the distribution and general movements of fin whales in the GoC is limited and comes primarily from the 1990s. Most recent publications have focused on genetics and acoustics, but do not address the movement patterns of fin whales within the GoC. The goal of this study is to fill this knowledge gap by describing fin whale movements and their inferred behavior in the GoC in space and time, using the first satellite telemetry data gathered on this supposedly resident population.

## Materials and methods

### Data

#### Ethics statement

Tagging occurred under the permit Oficio No. DOO 02–0427 from the Mexican Secretaría de Medio Ambiente y Recursos Naturales. The study also was conducted under U.S. National Marine Fisheries Service (NMFS) Permit No. 369–1440, authorizing close approach and deployment of implantable satellite tags on large whales, which are protected by the 1972 Marine Mammal Protection Act and the 1973 Endangered Species Act in the United States. All tagging procedures described in this permit, and used in this manuscript, were subjected to an internal NMFS and external review by veterinarians and other marine mammal researchers prior to approval. In addition, this study was carried out in strict accordance with the policies and guidelines of the Oregon State University Institutional Animal Care and Use Committee (IACUC), composed of veterinarians and other university administrators, under IACUC Permit No. 2284. IACUC acceptance assures that the research follows its guidelines for humane care and use while meeting its objectives to reduce, replace, and refine the use of animals in research.

### Study area

The GoC is a semi-enclosed sea adjacent to the Eastern North Pacific Ocean, renowned for its high productivity and biological diversity [40–44]. It is located between 107–115°W and 23–32°N, with a length of 1,100 km and a width ranging from 108 to 234 km [45]. The GoC is characterized by six basins with depths exceeding 2,000 m, and containing steep slopes, narrow and wide continental shelves, numerous islands, and coastal lagoons [46]. Two distinct seasons can be identified in the GoC: a highly productive cool season during the northern hemisphere's winter and spring, and a less productive warm season in the boreal summer and autumn [44,47,48].

### Satellite tag deployment

Fieldwork took place from 25–31 March 2001 in the southwestern GoC, along the east coast of the Baja California Peninsula, Mexico. Of 11 Argos-monitored radio tags, 10 were deployed on the east side of Carmen Island, off Loreto, and one was deployed in the Bay of La Paz. All tagged individuals were adults without calves and of unknown sex. Tags were deployed from a small vessel (~7 m) using a modified air-powered system [19,49], and placed as close to the midline of the body as possible. Tags were designed for nearly complete implantation into the blubber layer, and consisted of a Telonics ST-15 UHF Argos transmitter and two Duracell 2/3 A lithium batteries housed in a stainless steel cylinder (19 cm long by 1.9 cm in diameter). Details of tag design, construction, and attachment are described in Mate et al. [19]. To prolong battery life, the transmitters were programmed for continuous transmission for 4 hours every day during the time that satellites passed overhead for the first 90 days, and then for 4 hours every other day thereafter.

### Argos data processing

Tagged whales were tracked using the Argos satellite-based system that assigns a quality to each location, depending, among other things, on the number and temporal distribution of transmissions received per satellite pass (50). The accuracy associated with each Argos satellite location is reported as one of seven possible location classes (LC), in descending order of accuracy: 3, 2, 1, 0, A, B, Z and ranging from less than 200 m (LC 3) to greater than 5 km (LC B) [50,51]. Prior to analysis, Argos locations were filtered by LC as follows. First, locations of class Z were removed because of the unbounded errors associated with this class. Lower-quality LCs (LC 0, A, or B) were not used if they were received within 20 min of higher-quality locations (LC 1, 2, or 3). Finally, duplicate locations were discarded.

### Modeling

#### State-space modeling

The Bayesian SSM developed by Jonsen et al. [24–26] was applied to the filtered Argos locations for each track, using the R programming language v. 2.12.1 [52] and WinBUGS v. 1.4.3 [53]. The SSM is a time-series model that allows unobservable, true states to be inferred from observed data, while accounting for errors arising from imprecise observations and from stochasticity in the process being studied [23].

The model provided regularized tracks for each tagged whale with one estimated location per day, after accounting for Argos satellite location errors (based on Vincent et al., [51] and the movement dynamics of the animals. The SSM ran two Markov chain Monte Carlo (MCMC) simulations each for 30,000 iterations, where the first 10,000 iterations were discarded as a burn-in, and the remaining iterations were thinned by removing every fifth one [20,24]. This process left two 4,000-iteration long chains for analysis.

#### Analysis of movement behavior

Behavioral switching models classify animal movement behavior into coarse modes at temporal scales greater than the minimum observed sampling interval, based on fundamental differences in movement inferred from the observed locations. These tools are useful in identifying when and where animals engage in different activities (e.g., searching, foraging, resting, migrating [8,11,22].

Included in our SSM model implementation was the classification of locations into two behavioral modes: transiting (mode 1) and area-restricted searching (ARS; mode 2). These were based on mean turning angles (θ) and autocorrelation in speed and direction (γ). Even though only two behavioral modes were modeled, the posterior means of the MCMC samples provided continuous values between 1 and 2 [20,24]. As in Jonsen et al. [26] and Bailey et al. [20], we classified observations with posterior means greater than 1.75 as ARS behavior, and observations with posterior means lower than 1.25 as transiting behavior [21,22]. Locations with posterior mean values in between these cutoffs were labeled “uncertain” [24,26,27].

#### MCMC convergence assessment

Considering the small number of tagged individuals, we checked each track thoroughly to ensure all available tracks were accurately represented in our models for subsequent behavioral interpretation. We used the deviance and behavior parameters of MCMC convergence as indicator metrics to assess model convergence through a series of checks on the SSM output as follows:

1. The MCMC runs were visually verified for random travel along the sampling space and compared between the two generated chains [54,55].
2. Comparative trends in the cumulative averages and standard deviations for three parameters (deviance, θ, and γ) were visually examined to verify that the chains stabilized and to compare the relative performance between the two chains.
3. The resulting distributions of each parameter were visually inspected to identify anomalies like bimodality or incomplete tails, which would indicate departures from normality [56].

To more thoroughly assess the performance of each modeled track, we derived six additional metrics from the Argos data: a) the mean number of filtered locations per day, b) the standard deviation of the number of filtered locations per day, c) the input:output ratio of Argos:SSM locations, d) the total number of tracking days, e) the total number of Argos locations filtered, and f) the ratio of total number of Argos locations filtered to the total number of tracking days. We used simple linear regression modeling in the R software to determine whether any of these track-level metrics influenced the respective deviance (i.e., -2 × log-likehood + a standardizing factor) from the SSM [54,55,57].

### Post-processing

#### SSM locations occurring on land

Due to the long and narrow geometry of the GoC as well as the presence of numerous islands, a number of estimated SSM locations occurred on land (since the SSM method is not land aware). To ensure the SSM tracks did not cross over land, we developed an ad-hoc method to move these locations over water. For this purpose, we used the 95% credible limits in longitude and latitude provided by the SSM respectively as the semi-major and semi-minor axes of an ellipse around each land-based SSM location (with the premise that a location can fall anywhere inside this ellipse with 95% probability) through a custom script in Python v.2.7 [58,59] For each ellipse, the land-based portion was deleted and the centroid of the remaining ocean-based portion was calculated and replaced for each land location in the track, while retaining the behavioral mode originally estimated for the land-based locations by the SSM. A final set of SSM tracks was built after the land-based locations were corrected, from which speed and distance between location pairs were calculated by behavioral mode in Python [59].

#### Seasonality

The ocean surface conditions of the GoC are dominated by atmospheric forcing and ocean dynamics. Higher temperatures are found at the head and the mouth of the gulf during the summer while lower temperatures are found in the northern half and around the Midriff Islands throughout the year. As a result, two climatic seasons exist: a cool season from December to May and a warm season from June to November [41,44,48,60–63]. Considering this, the resulting tracks were split according to the timing of the cool and warm seasons to understand how fin whale movements were distributed seasonally in time and space. Maps of the movements and inferred behavioral mode where created for each season in ArcGIS v.10.3 [64].

## Results

A total of 11 satellite tags were deployed in 2001. One tag (PTT 23033) did not send any valid location and another one (PTT 10840) only transmitted five locations, so both were excluded from further analyses. The remainder of the tags (n = 9) transmitted a total of 607 locations (after excluding LC Z). Mean track duration was 70 days (± SD 61 days). The mean number of locations per day was 1.7 (± SD 0.6 locations per day) (Table 1).

**Table 1:** Summary of tracking data for 11 Argos-monitored radio tags deployed on fin whales in the Gulf of California in 2001. For each SSM modeled track, D is the deviance and DIC is the deviance information criterion.

PTT	Transmission date		Tracking days	Number of locations		SSM results	
	Deployed	Last message		Total Locs	Locs/day	D	DIC
**829**	2001-Mar-26	2001-Apr-30	35	8	1.2	-184	-179
**830**	2001-Mar-31	2001-May-27	57	32	1.3	-64.3	-19.7
**849**	2001-Mar-30	2001-Sep-22	176	242	1.9	-1176	-922
**824**	2001-Mar-31	2001-Apr-20	20	51	2.6	-51.4	-20.1
**833**	2001-Mar-27	2001-Apr-28	32	78	2.6	-188	-139
**834**	2001-Mar-22	2001-Apr-11	20	13	1.1	-111	-79.5
**836**	2001-Mar-26	2001-Aug-26	153	50	1.1	62.92	126.1
840*	2001-Mar-26	2001-Aug-05	10	5	NA	NA	NA
**843**	2001-Mar-26	2001-Apr-21	26	31	2.1	-143	-111
23033*	2001-Mar-31	2001-Apr-10	10	0	NA	NA	NA
**23038**	2001-Mar-27	2001-Jul-23	118	96	1.5	-542	-390

* These tags were excluded from analysis due to too few data points.

### MCMC convergence assessment

The distribution of the deviance and the movement parameters (θ and γ) in the MCMC chains indicated that these parameters were generally well-behaved for all tracks except PTT 829, which only had eight Argos locations (see S1 File).

**Fig 1:**
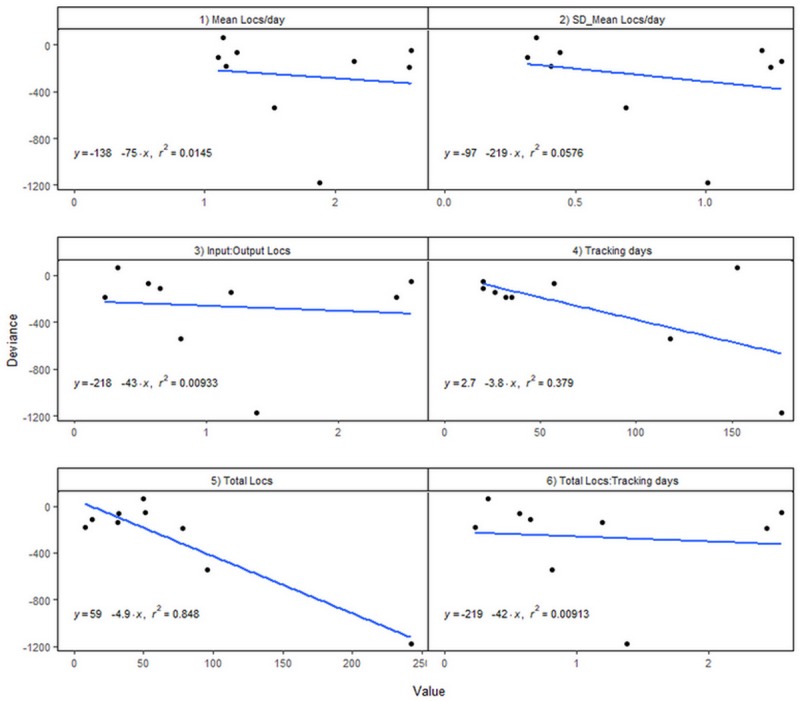
The relationship between the deviance and the derived per-track metrics. a) mean number of locations (Locs) per day; b) SD of mean number of locations per day; c) the ratio of Argos:SSM locations; d) total number of tracking days; e) total number of locations per track; and f) the ratio of total number of Argos locations to the total number of tracking days.

The linear regression of the deviance on the derived track metrics indicated that only the total number of locations per track had a reasonable explanatory power on the deviance value (R^2^ = 0.847, F = 38.95, p < 0.0004). The other metrics did not have a significant relationship to the deviance (Fig 1). As a result, the SSM output for PTT 829 was discarded (see S1 File).

**Fig 2:**
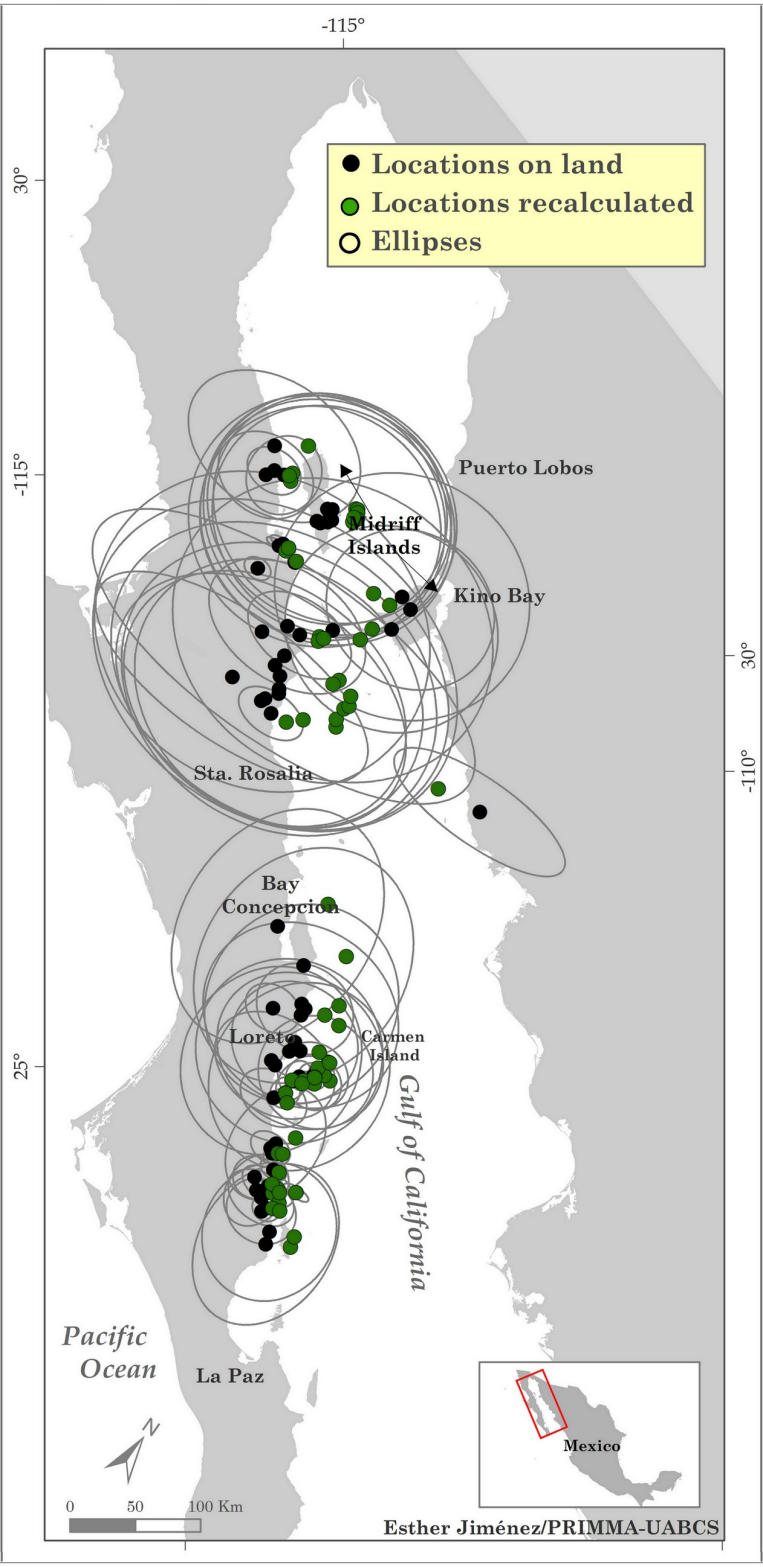
Correction of land-based SSM locations. Ellipses representing the SSM 95% credible limits in longitude and latitude are shown in black. Black dots are locations on land, and green dots are the respective corrected locations.

The remaining eight good Argos tracks generated 562 SSM locations, of which 11% occurred on land (Fig 2).

### Behavioral inferences

**Fig 3:**
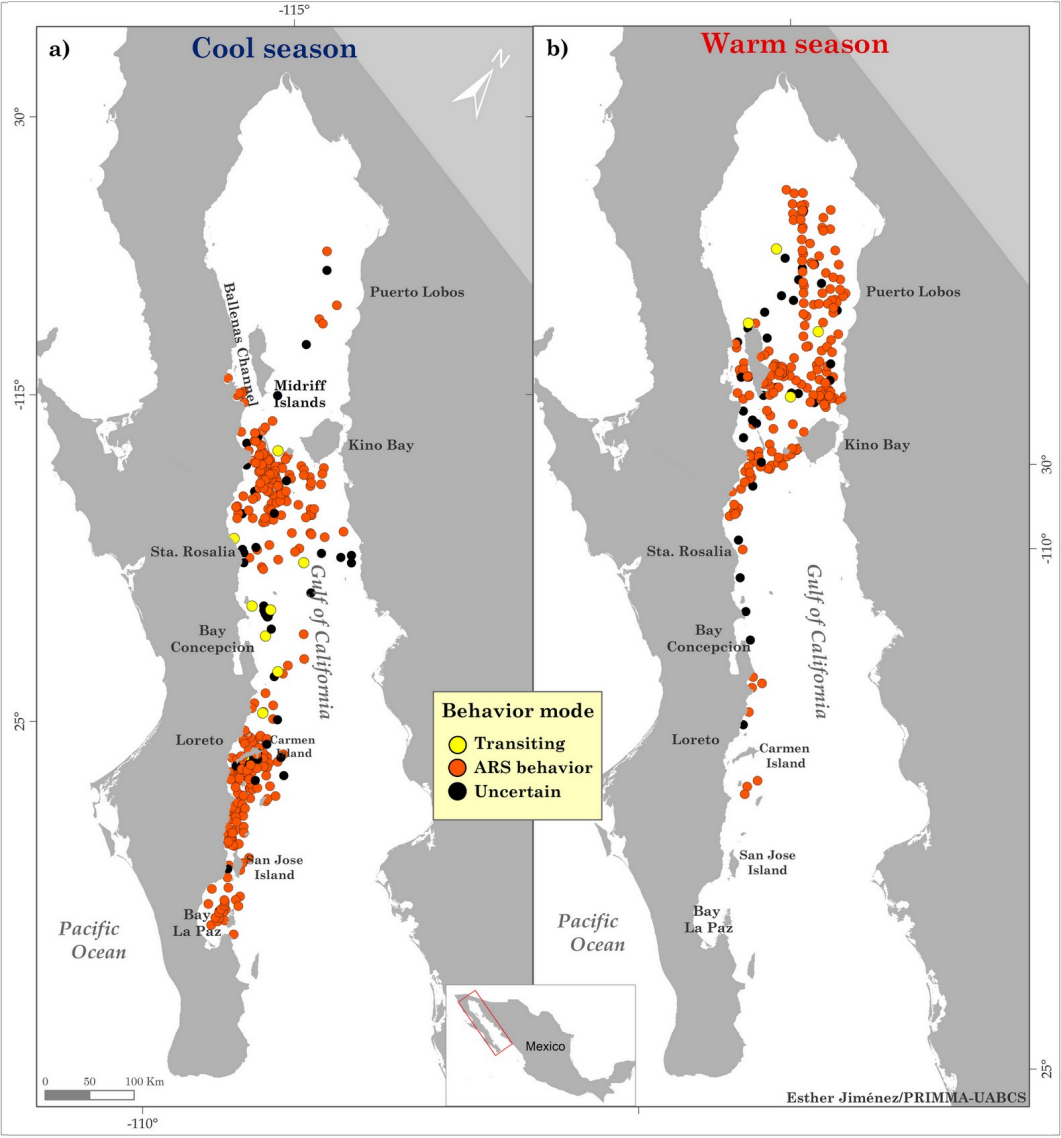
Spatial distribution of the classification of behavioral mode by season for fin whale SSM locations. Left is the cool season (from December to May; n = 333) and right is the warm season (from June to November; n = 229).

Behavioral modes inferred by the SSM indicated that ARS behavior was dominant for both the cool (83% of the SSM locations) and the warm (84% of the SSM locations) seasons during the monitoring period. Transiting behavior occurred for 3–4% of the SSM locations and uncertain behavior corresponded to 14% of the SSM locations for each season. Spatially, ARS behavior during the cool season was located mainly in two areas: 1) Loreto-La Paz Corridor, where the inshore habitats were the principal habitat for fin whales, and 2) the area between Santa Rosalia and the southern Midriff Islands. The space in between areas (1) and (2) contained tracks of fin whales exhibiting uncertain and transiting modes, indicating that the whales were not using these areas for long periods, but merely passing through (Fig 3, left). During the warm season, tracking data showed the preferred areas were in the center of the GoC, with ARS behavior occurring around Midriff Islands and along the coast of Sonora State (from Tiburon Island to Puerto Peñasco). Transiting and uncertain behaviors were also exhibited around the Midriff Islands (Fig 3, right).

On average, fin whales traveled 25 km per day (range: 22.94–28.31 km), with a mean speed of 1.06 km/h. The minimum total tracked distance was 1,034 km and the maximum was 13,199 km (Table 2). During ARS mode, travel speed was slower (mean = 0.84 km/h) than during transiting mode (mean = 3.48 km/h).

**Table 2:** Mean distances and speeds traveled between consecutive daily SSM locations for eight fin whales tracked in the Gulf of California in 2001.

PTT	Distance (km)					Speed (km/h)				Speed by behavioral mode (km/h)		
	Mean	SD	Min	Max	Total track	Mean	SD	Min	Max	Transiting	ARS	Uncertain
**824**	27.89	22.8	0.66	115.4	6136.6	1.16	0.95	0.03	4.81	NA	1.01	0.25
**830**	23.51	14.68	2.57	64.68	1034.55	0.98	0.61	0.11	2.69	NA	0.96	1.58
**833**	27.34	22.06	0.66	115.4	6861.25	1.14	0.92	0.03	4.81	NA	0.96	0.21
**834**	26.33	21.76	0.66	115.4	7108.39	1.1	0.91	0.03	4.81	NA	0.5	1.18
**836**	23.38	21.36	0.07	129	9867.44	0.97	0.89	0	5.38	5.09	0.66	0.06
**843**	22.94	21.18	0.07	129	10252.9	0.96	0.88	0	5.38	ND	0.65	0.37
**849**	28.31	23.29	0.66	115.4	5718.87	1.18	0.97	0.03	4.81	2.54	1.1	0.55
**23038**	23.49	21.23	0.07	129	13199.65	0.98	0.88	0	5.38	2.5	0.88	0.03
**Mean**	25.4		0.68	114.16	7522.46	1.06		0.03	4.76	3.38	0.84	0.53

### Seasonality

**Fig 4:**
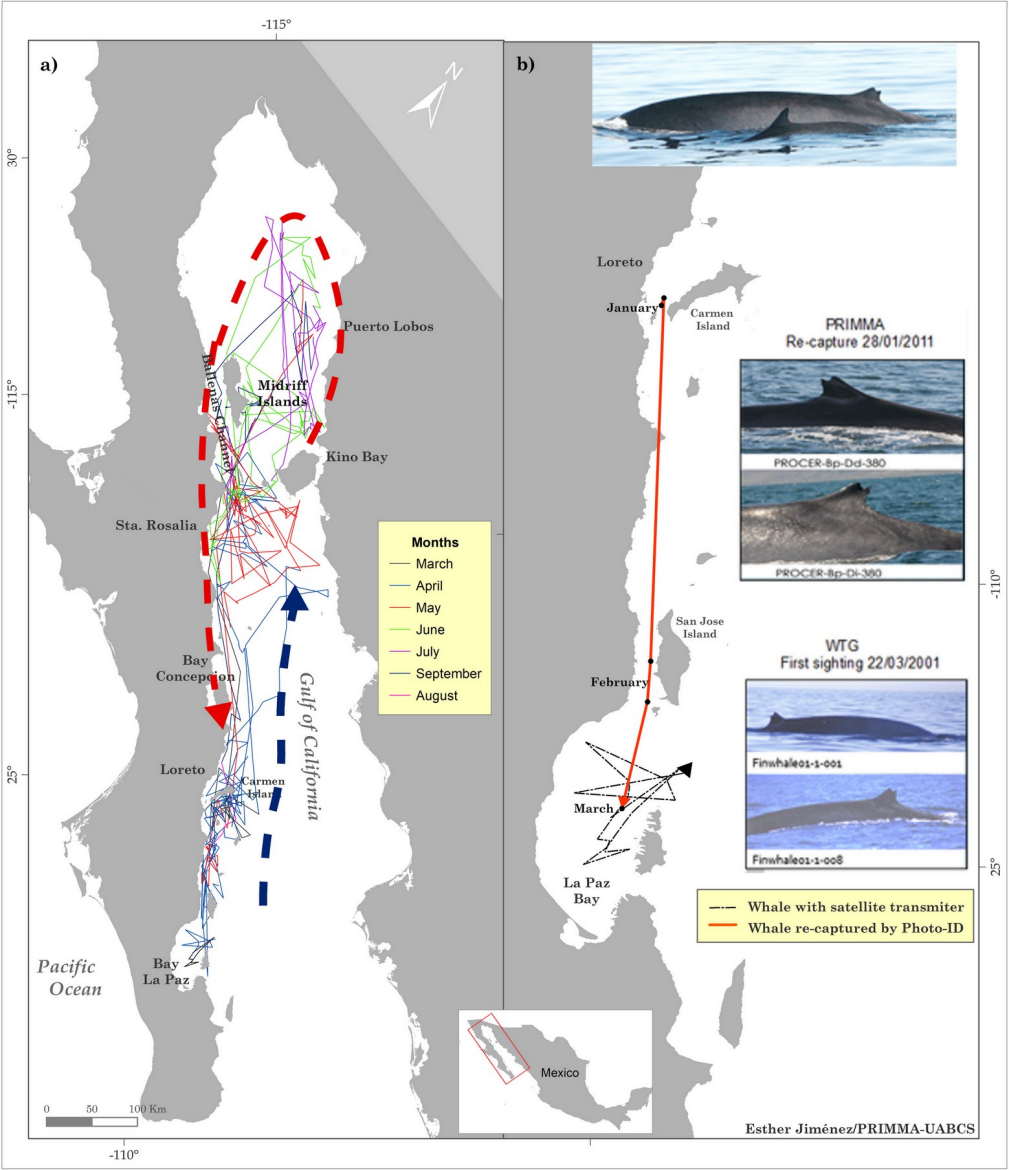
a) Seasonality of movements of eight fin whales tagged in March 2001 from SSM-derived locations. Portions of the tracks associated to specific months, the dashed lines represent the purported migratory pattern; b) the black line indicates the whale with tag PTT 10834 tagged and photographed in the Bay of La Paz by the Whale Telemetry Group (WTG), Marine Mammal Institute, Oregon State University during 2001. The orange line indicates the photographic re-capture location of this whale in 2011 by Programa de Mamíferos Marinos (PRIMMA/UABCS), Universidad Autónoma de Baja California Sur, Mexico, in the company of a calf.

All the tags transmitted into the cool season. Two whales (PTT 10824 and 10833) moved between Loreto and San Jose Island during March and April and then their tags stopped transmitting. The individual tagged in the Bay of La Paz (PTT 10834) stayed in the area until April, when the tag stopped transmitting. The individual with the PTT 830 moved northward towards Santa Rosalia, where that tag also stopped transmitting. The rest of the whales (PTT 849, 10836 and 23088) migrated northward between April and May along the western margin of the GoC, and continued to send data up until the warm season (Fig 4A).

During the warm season, only three individuals moved northward past Santa Rosalia and into the upper GoC through the Midriff Islands. These tags lasted for more than 100 days into the cool season and just one whale (PTT 849) moved southward in the GoC, ending up off Loreto (Fig 4A; see S2 File for individual maps of each track). A photograph of the whale with tag PTT 10834 (tagged in Bay of La Paz) was taken by the Whale Telemetry Group (WTG) at Oregon State University’s Marine Mammal Institute at the moment of tagging (in 2001), and it was matched with the fin whale photographic catalog from Programa de Investigación de Mamíferos Marinos from the Universidad Autónoma de Baja California Sur (PRIMMA/UABCS). The matching photograph, collected in the nearby San Jose Channel, was dated 28 January 2011 (10 years later), in which the whale was accompanied by a calf, evidence that the tagged individual was female (Fig 4B).

## Discussion

### MCMC convergence assessment

SSM analysis of animal telemetry data has been used in various ways, including to filter error-prone Argos [50] and light-based [65] locations, or to estimate unobserved behavioral states (e.g., 27,30). Indeed, SSMs have become a prominent tool in the study of animal movement [66]. However, SSMs cannot always deal with some of the issues inherent to telemetry data from marine animals, like temporal gaps in the reception of Argos locations, tracks of short duration, or unclassifiable movement behavior. Exploration of the MCMC convergence and the distribution of the deviance and the behavior parameters, was a useful way to decide to keep or discard tracks with small numbers of locations. Tracks with few Argos locations that were spread over many days led to problems of stability and convergence in all MCMC parameters. This information was confirmed by observed departures from normality of the distribution of the deviance and the behavior parameters and by the regression analyses. For this reason, the transmission schedule programmed into Argos-monitored tags needs to weigh battery preservation considerations against performance in analytical methods like SSMs to avoid erroneous interpretations about movement and inferred behavior.

### Movements and behavioral inference

The present study represents the first attempt to examine the movements and behavior of fin whales in the GoC through satellite telemetry. Despite the small sample size, the information on movement data is valuable for understanding how fin whales move, use, and connect the areas along the GoC. Our findings support the general movements in time and space previously described based on photo-identification studies in the GoC. They also support the previous notion that the population does not leave the GoC, and contributes to fine-scale knowledge about the extent to which fin whales use their habitat. This information could provide the baseline for developing conservation and management efforts for this resident population.

We identified two principal destinations for fin whales in the GoC for their seasonal migration, the Loreto-La Paz Corridor and the Midriff Islands, which both showed a high percentage of ARS behavior (83–84%) for each season. ARS behavior mostly has been related to feeding activities due to the animals occupying patchily distributed areas of sufficiently abundant prey. This is because shifting between the patches increases search effort and thus decreases the likelihood of securing enough food [66]. During the cool season, fin whales travel northward along the western margin of the GoC, where environmental conditions are dominated by a combined north-south current [44,47,48,67] and outgoing tidal currents acting to aggregate euphausiids (*Nyctiphanes simplex*), which are the principal prey for fin whales in the GoC [68–70]. This season is also the breeding season for the euphausiids, producing more than 80% of the calyptopis larval phase in the mid-southern GoC at Carmen Island (off Loreto), San Jose Island, and the Baja California Peninsula [69,71,72]. In contrast, during the warm season, this area is mainly where the food concentrations fall due to temperature changes.

The seasonal movements showed the fin whales reduced their activity and spent time in certain habitats in the GoC. Even though there is a general lack of knowledge about how fin whales use the GoC, most of the data published refers to the Midriff Islands, where fin whales have been observed in feeding activity. However, the species could have a wider distribution to the south [39,71,73,74]. The results of this study effectively show that there are more places than in the northern GoC where fin whales are distributed during the cool season than had been previously documented. Also, in these newly observed areas, the whales exhibited the same types of behavior as in the northern feeding areas [39,71,75]. Our results are in broad agreement with seasonal movements mentioned by other authors who coincidentally recorded fin whales in the Loreto-La Paz Corridor [36] with high prey biomass known to be important for fin, blue (*Balaenoptera musculus*), and Bryde's whales (*Balaenoptera edeni*) [69,76–78]. This habitat overlap during the cool season is strongly driven by the prey species, even though these whale species are all in this krill-based foraging ground during winter and spring [20,79].

On the other hand, it is important to note that December to February is the reproductive season for fin whales. Even though the ARS mode behavior is assumed to represent feeding activity, it could also be a proxy for other social interactions [66] that relate to reproductive behavior since the data are being collected in seasonally reproductive areas.

Concerning the warm season, the tagged whales moved to the northern GoC and ARS behavior also occurred in this area, especially in the vicinity of the Midriff Islands. We suggest this movement is also related to prey distribution because this area is one of the two places (Bay of La Paz being the other place) in the GoC where euphasiids can avoid the highest temperatures of the GoC during the summer. By the end of the summer, their highest densities start to fall [80,81]. Some authors have suggested that to deal with the decline in euphausiids, fin whales exploit other potential seasonal prey source [71]. While the euphasiids decrease in biomass, that of the Pacific sardine (*Sardinops sagax*) increases in this area, [82] and during the autumn the sardines migrate to the south to Loreto along the western margin of the GoC. Through isotope analyses, this hypothesis has been investigated, finding that fin whales switch prey during the summer, resulting in an increase in δ15N for the euphausiid *Nyctiphanes simplex* in skin samples during the summer [83]. Comparing skin samples from fin and Bryde´s whales the similarity in δ15N values also coincides with the known icthyophagous habits of the Bryde’s whale [84]. Considering all this, we suggest that fin whale movements in the GoC are adapted in time and space to coincide with high densities of both euphausiid and fish prey types.

For the warm season, we suggest the main activity of fin whales, as evidenced by ARS behavior distributions, is indicative of foraging activity. At this time, their distribution is consistent with the distribution of prey species as described by other authors [33]. Also, the tracking data collected during the warm season was outside the mating and calving season for this species [85–87]. Similar behavior has been documented for other resident fin whale populations such as the Mediterranean population, which primarily exhibits ARS behavior throughout the year as they move between potential feeding areas [31,88,89]. These cases are not unique among balaenopterids, as there are other non-migratory populations found in the tropics, subtropics, and enclosed seas, such as Bryde’s whales [76] and one distinct population segment of humpback whales (*Megaptera novaeangliae*) in the Arabian Sea [88], which is able to subsist on year-round productivity.

The satellite telemetry data obtained in this study revealed five important details about movements fin whales in the GoC: 1) the tagged individuals did not move toward the mouth, much less leave the GoC, supporting the hypothesis of its residence. 2) The movements of the tagged whales were related in time and space to the distribution of their prey. 3) Fin whale movement behavior was consistent with foraging activity year round, as is the case for the isolated population in the Mediterranean Sea [90]. 4) No tagged whales moved to the eastern margin of the GoC to the south of the Midriff Islands (this was only observed in the upper GoC). 5) The photographic recapture of the whale with tag PTT 10384 10 years later in the company of a calf in the nearby San Jose Channel, suggests seasonal site fidelity to the southern GoC and a possible calving area during the cool season.

Finally, the fin whale movements documented in this study overlapped with anthropogenic activity in the GoC, mainly traffic by fishing and tourism vessels [91]. The main risks of this overlap include entanglements in fishing gear, and harassment by boats that approach too closely [92]. The intensity and spatial extent of these threats to marine mammals in the GoC is unclear, but sightings of entangled cetaceans or individuals with ship strike marks have been increasing in recent years in Mexico [91]. The results obtained in this study will help better identify areas where interactions between fin whales and threats need to be monitored and evaluated to make better decisions regarding development plans and to manage responses to human activities [93]. Also, we suggest a greater sampling effort during the warm season in the north of the GoC to gain a better understanding of fin whale movements during this time for which data remain scarce.

## Supporting information

S1 File Convergence diagnostics for the MCMC deviance and for the behavioral parameters. (PDF)

S2 File The SSM tracks for eight fin whales tagged in March 2001 in the southwestern Gulf of California and their behavioral mode. (PDF)

## Abbreviations

ARS: area-restricted searching
d: day
DIC: Deviance Information Criterion
GoC: Gulf of California
LC: location class
Locs: locations
MCMC: Markov chain Monte Carlo
PTT: Platform Terminal Transmitter
SSM: state-space model
