# Supplementary material for: Fin whale movements in the Gulf of California, Mexico, from satellite telemetry
Source: PLoS One. 2019 Jan 10;14(1):e0209324. doi: 10.1371/journal.pone.0209324 (PMC6328206; doi:10.1371/journal.pone.0209324)
Supplement: S1 File — (PDF) [file pone.0209324.s001.pdf]

Additional File 1

Convergence diagnostics for the MCMC deviance

On pages 1 to 6 of this document, we present convergence diagnostics for the MCMC deviance ($\bar{D}$) for each SSM track. On each page, panel A contains the MCMC runs for the two chains (blue and red). Panels B and C show the cumulative average and the cumulative standard deviation for the two chains of the deviance, respectively. Panels D and E show the frequency histograms of the posterior distributions for each chain.

Notes for the deviance:

- While for most PTTs the deviance appeared to travel the sampling space (*y*-axis) in shorter periods, for PTT 829 (p. 2) the chains remained in a portion of the sampling space for longer.
- The frequency histograms of the posterior distributions for PTT 829 appeared very flat, long-tailed, and even bimodal for one of the chains, unlike the rest of the PTTs in the data set.

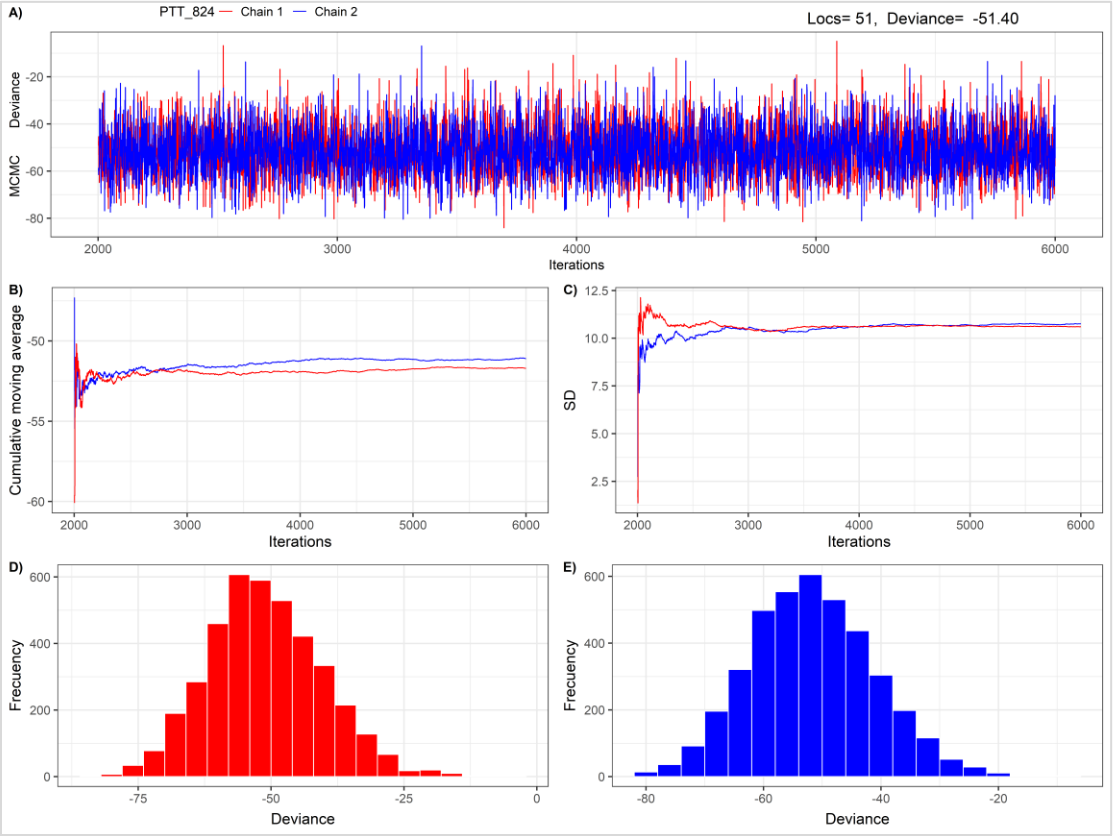
A)
PTT_824 Chain 1 Chain 2
Locs= 51, Deviance= -51.40
MCMC Deviance
Iterations
B)
Cumulative moving average
Iterations
C)
SD
Iterations
D)
Frecuency
Deviance
E)
Frecuency
Deviance

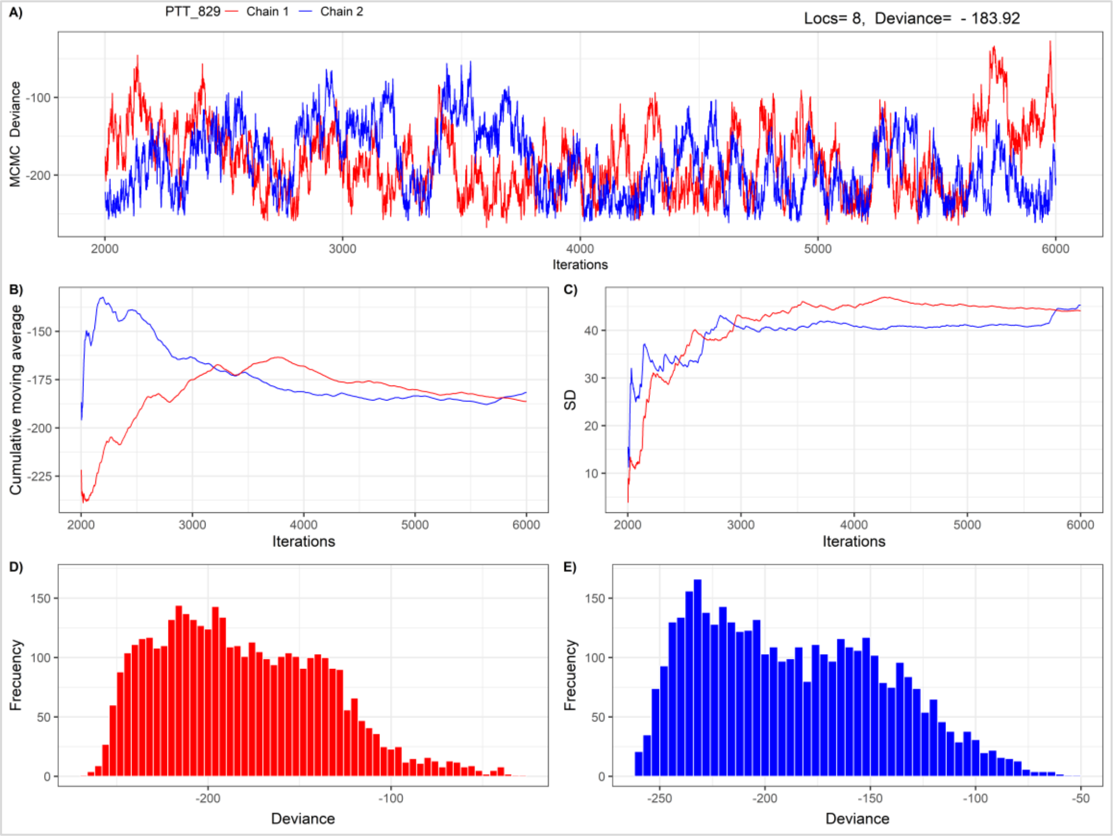
A)
PTT_829 Chain 1 Chain 2
Locs= 8, Deviance= - 183.92
MCMC Deviance
Iterations
B)
Cumulative moving average
Iterations
C)
SD
Iterations
D)
Frecuency
Deviance
E)
Frecuency
Deviance

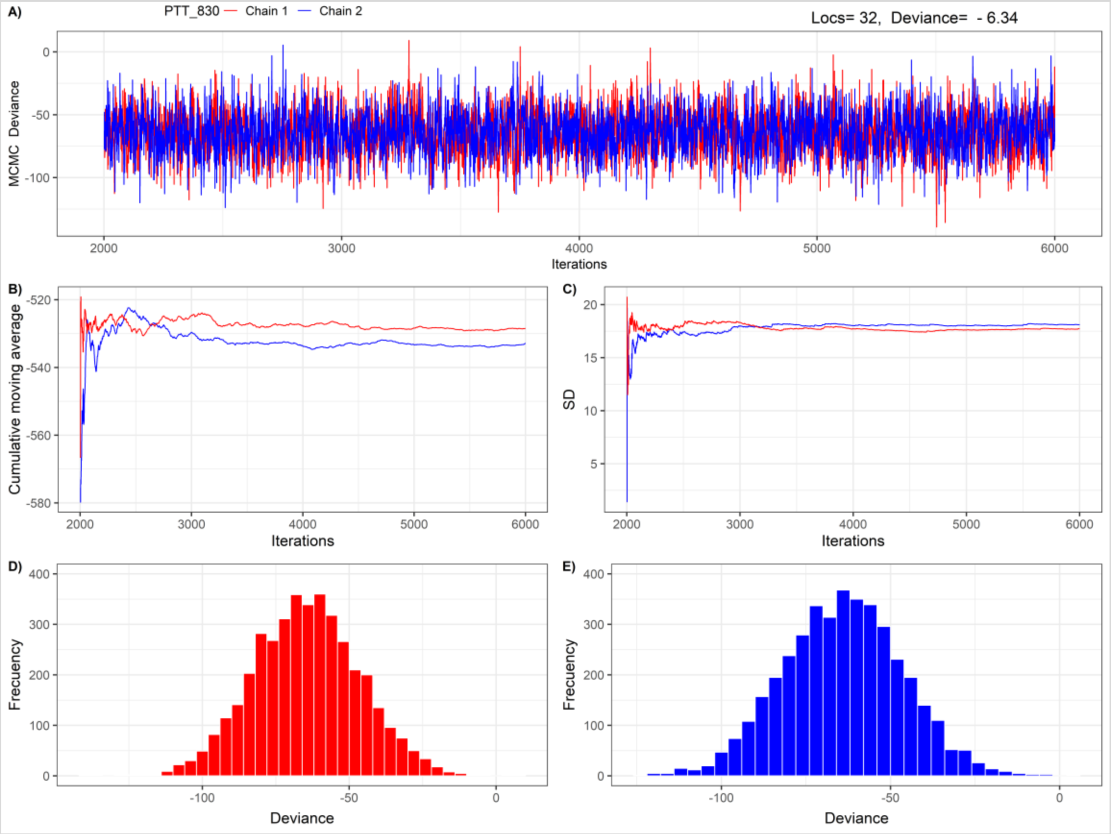
A)
PTT_830 Chain 1 Chain 2
Locs= 32, Deviance= - 6.34
MCMC Deviance
Iterations
B)
Cumulative moving average
Iterations
C)
SD
Iterations
D)
Frecuency
Deviance
E)
Frecuency
Deviance

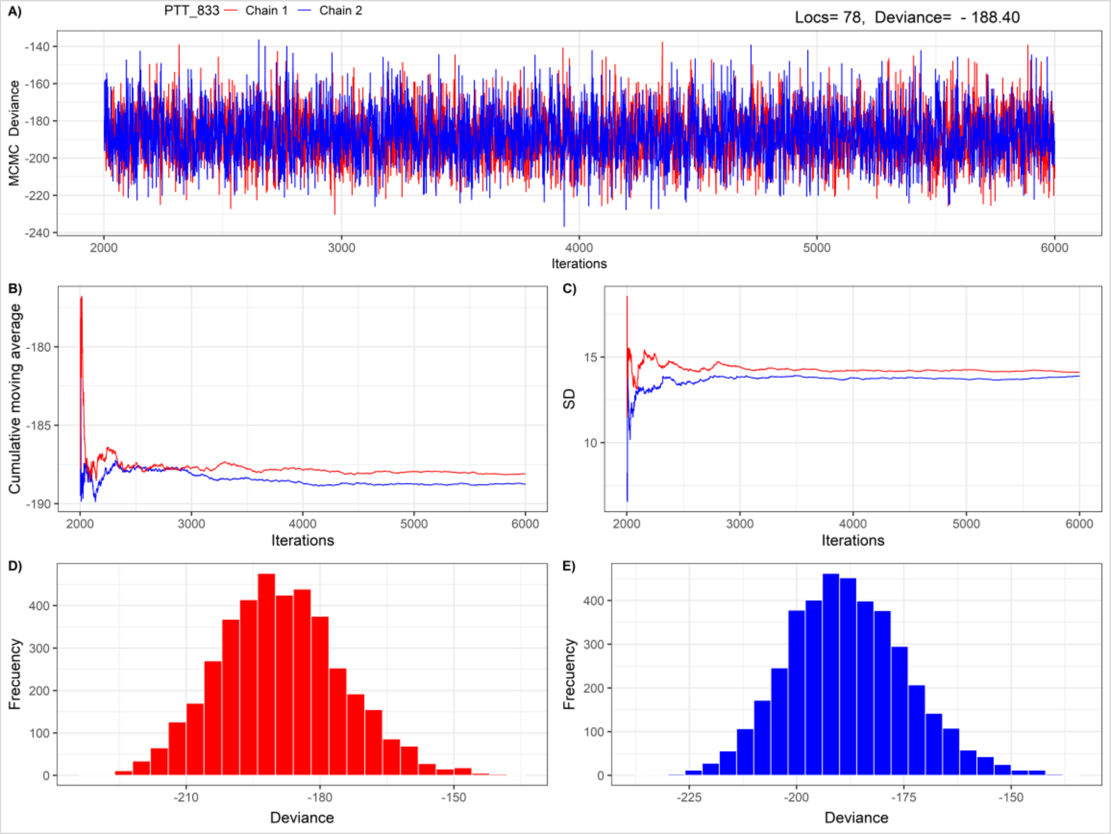
A)
PTT_833 Chain 1 Chain 2
Locs= 78, Deviance= - 188.40
MCMC Deviance
Iterations
B)
Cumulative moving average
Iterations
C)
SD
Iterations
D)
Frecuency
Deviance
E)
Frecuency
Deviance

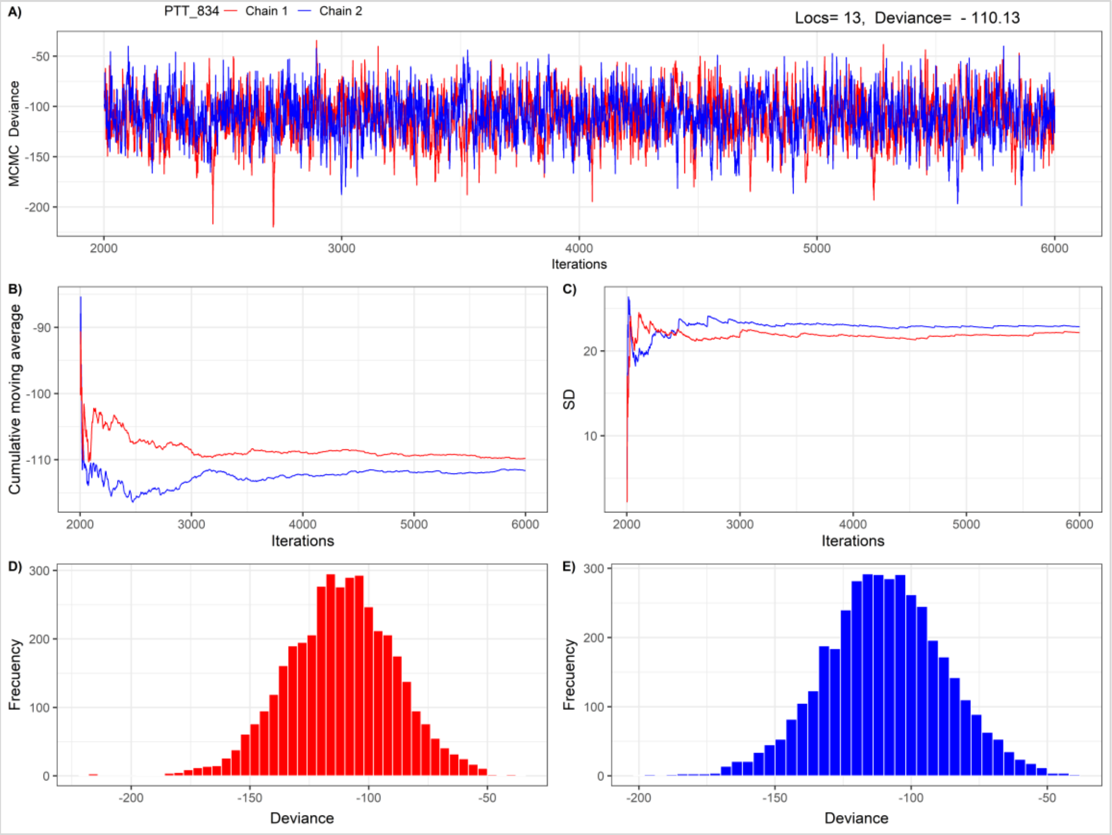
A)
PTT_834
Chain 1
Chain 2
Locs= 13, Deviance= - 110.13
MCMC Deviance
Iterations
B)
Cumulative moving average
Iterations
C)
SD
Iterations
D)
Frecuency
Deviance
E)
Frecuency
Deviance

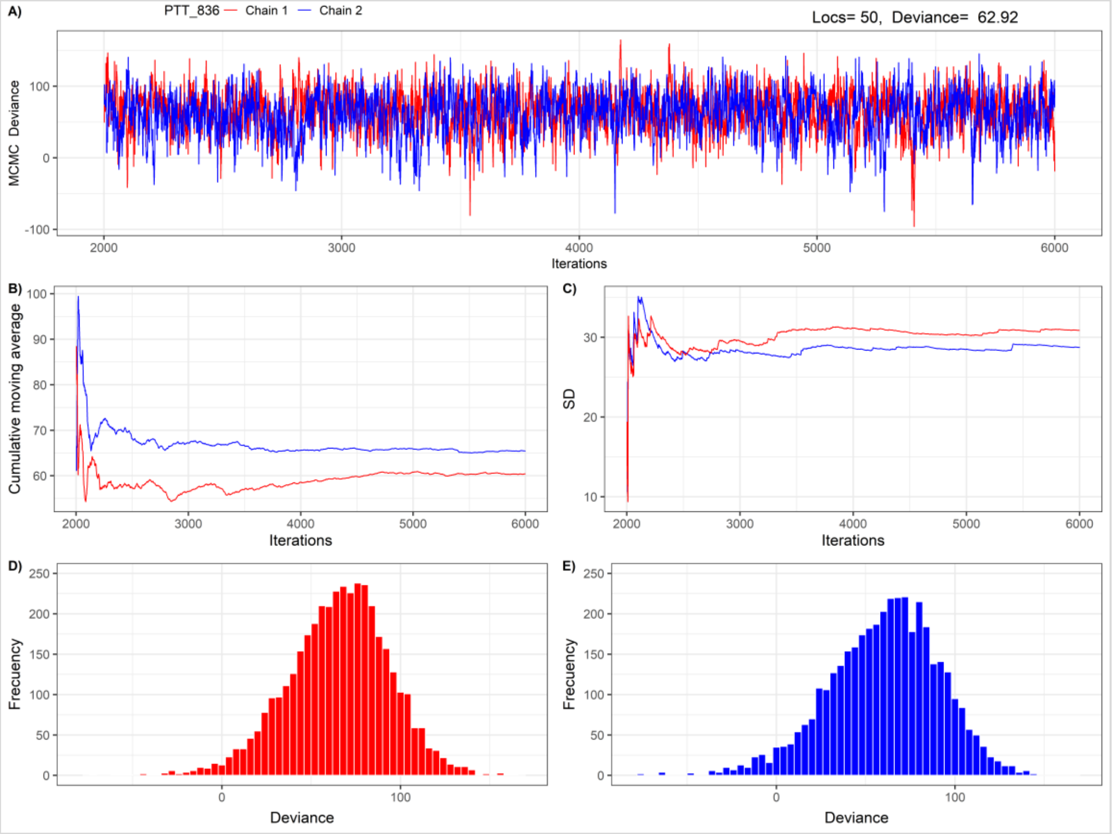
A)
PTT_836
Chain 1
Chain 2
Locs= 50, Deviance= 62.92
MCMC Deviance
Iterations
B)
Cumulative moving average
Iterations
C)
SD
Iterations
D)
Frecuency
Deviance
E)
Frecuency
Deviance

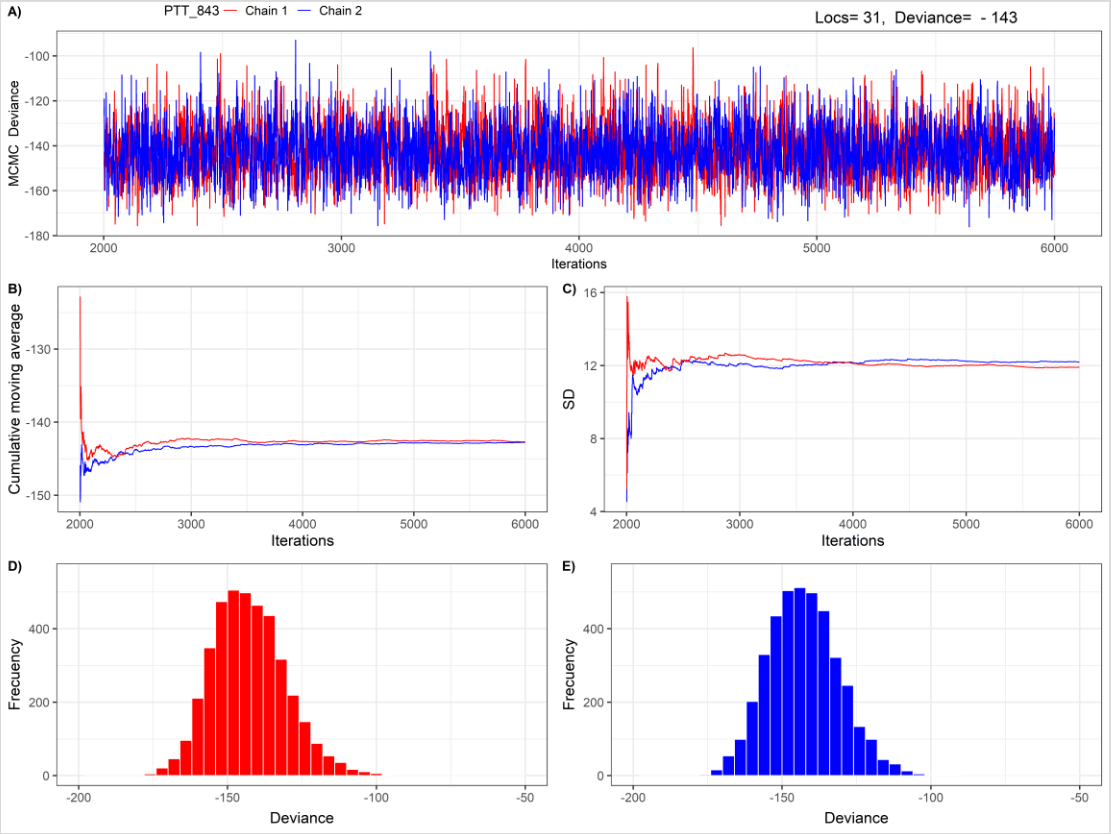
A)
PTT_843 — Chain 1 — Chain 2
Locs= 31, Deviance= - 143
MCMC Deviance
Iterations
B)
Cumulative moving average
Iterations
C)
SD
Iterations
D)
Frecuency
Deviance
E)
Frecuency
Deviance

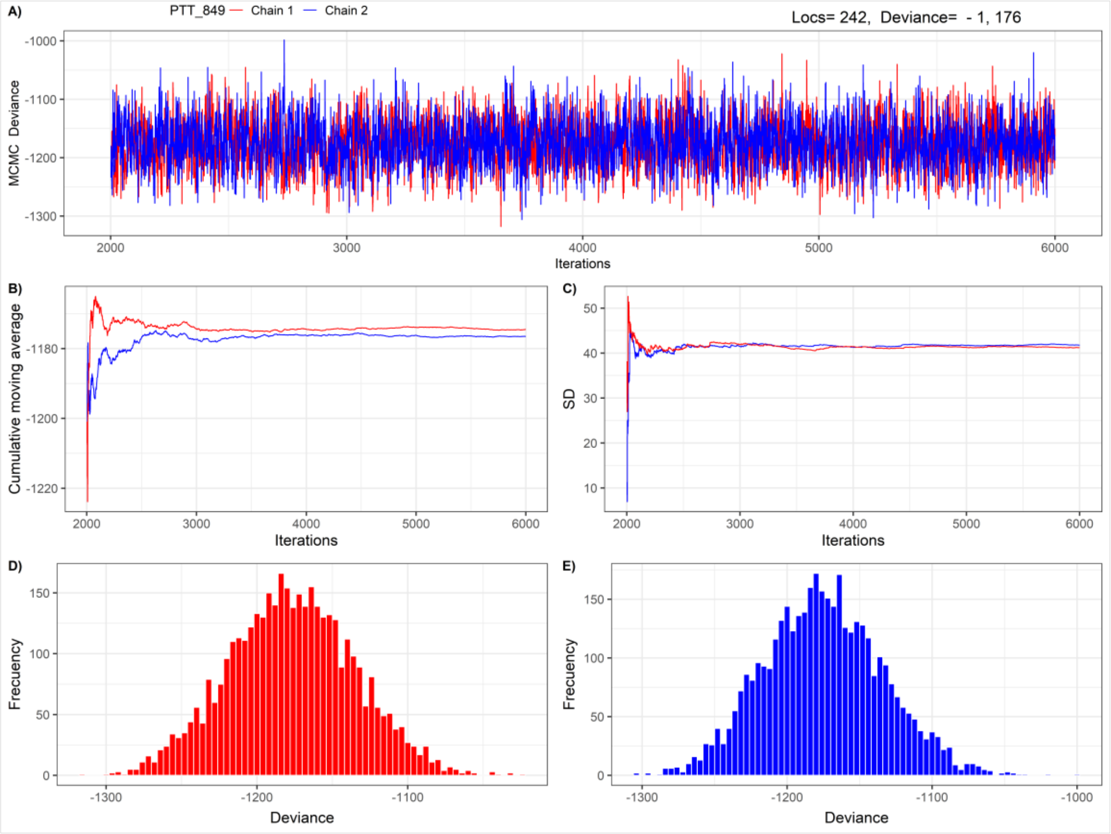
A)
PTT_849 — Chain 1 — Chain 2
Locs= 242, Deviance= - 1, 176
MCMC Deviance
Iterations
B)
Cumulative moving average
Iterations
C)
SD
Iterations
D)
Frecuency
Deviance
E)
Frecuency
Deviance

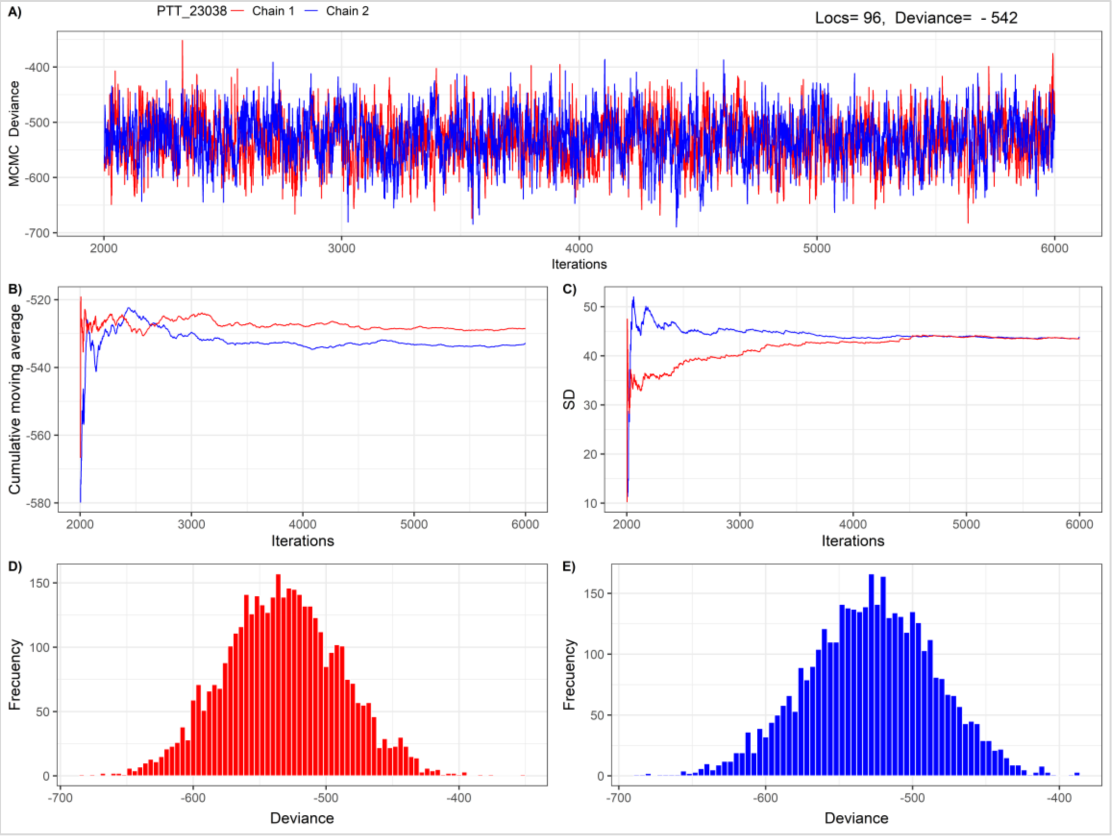
A)
PTT_23038 Chain 1 Chain 2
Locs= 96, Deviance= - 542
MCMC Deviance
Iterations
B)
Cumulative moving average
Iterations
C)
SD
Iterations
D)
Frecuency
Deviance
E)
Frecuency
Deviance

Convergence diagnostics for the behavioral parameters

On pages 7 to 25 of this document we present convergence diagnostics for the two behavioral parameters, θ (turning angles) and γ (autocorrelation in speed and direction), for each SSM track. On each page, panel A) contains the parameter's MCMC run for the two chains; panel B) contains the cumulative mean average (CMA) for the two chains; panel C) contains the cumulative standard deviation; and panels D) and E) the frequency histograms of the posterior distributions for the two MCMC runs. Note that the *x*-axes for the MCMC runs (first row in all plots) are on the same scale to allow an easy comparison. The same is the case for the *x*-axis on the frequency histograms.

<u>Notes for the behavioral parameters:</u>

PPT 829 showed some differences with respect to the rest of the SSM tracks in the data set, as follows:

- While for most PTTs the value of the parameter appeared to travel the sampling space (*y*-axis) in shorter periods, for PTT 829 the chains remained in a portion of the sampling space for longer (p. 9).
- The cumulative averages and the cumulative standard deviations tended to stabilize at least from the middle of the run (for some PPTs this occurred very close the beginning). On the contrary, for PTT 829 they appeared more unstable, and for the cumulative standard deviation of Theta 2 they were still changing at the end of the run (p. 9).
- The frequency histograms of the posterior distributions for PPT 829 look skewed and with some irregularity between class intervals (p. 9).
- For PPT 849, the frequency histogram of the posterior distribution of Theta 1, Chain 2, also looks somewhat unusual, but this is not extremely problematic (p. 15).

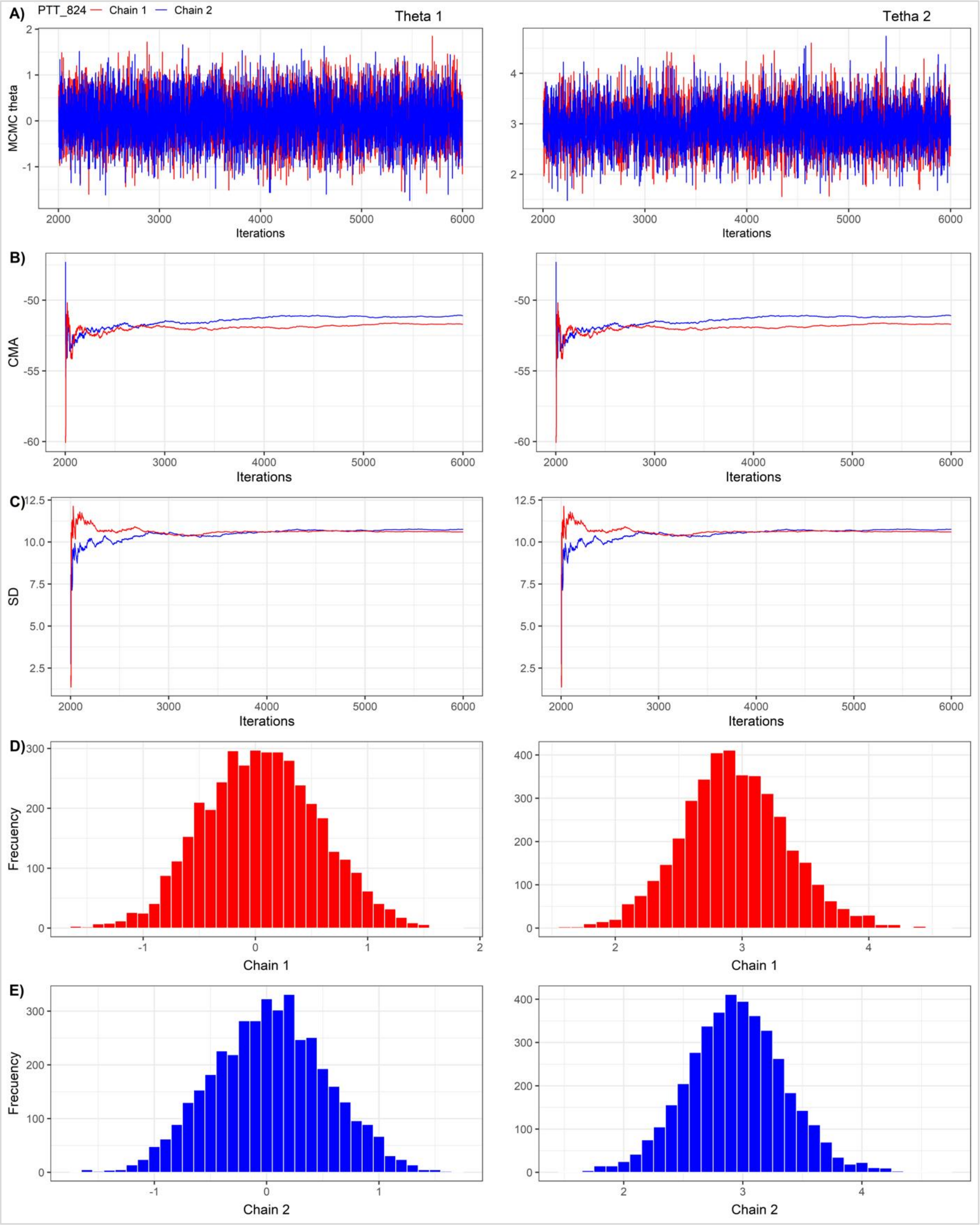
A)
PTT_824 Chain 1 Chain 2
Theta 1
Tetha 2
MCMC theta
Iterations
B)
CMA
Iterations
C)
SD
Iterations
D)
Frecuency
Chain 1
E)
Frecuency
Chain 2

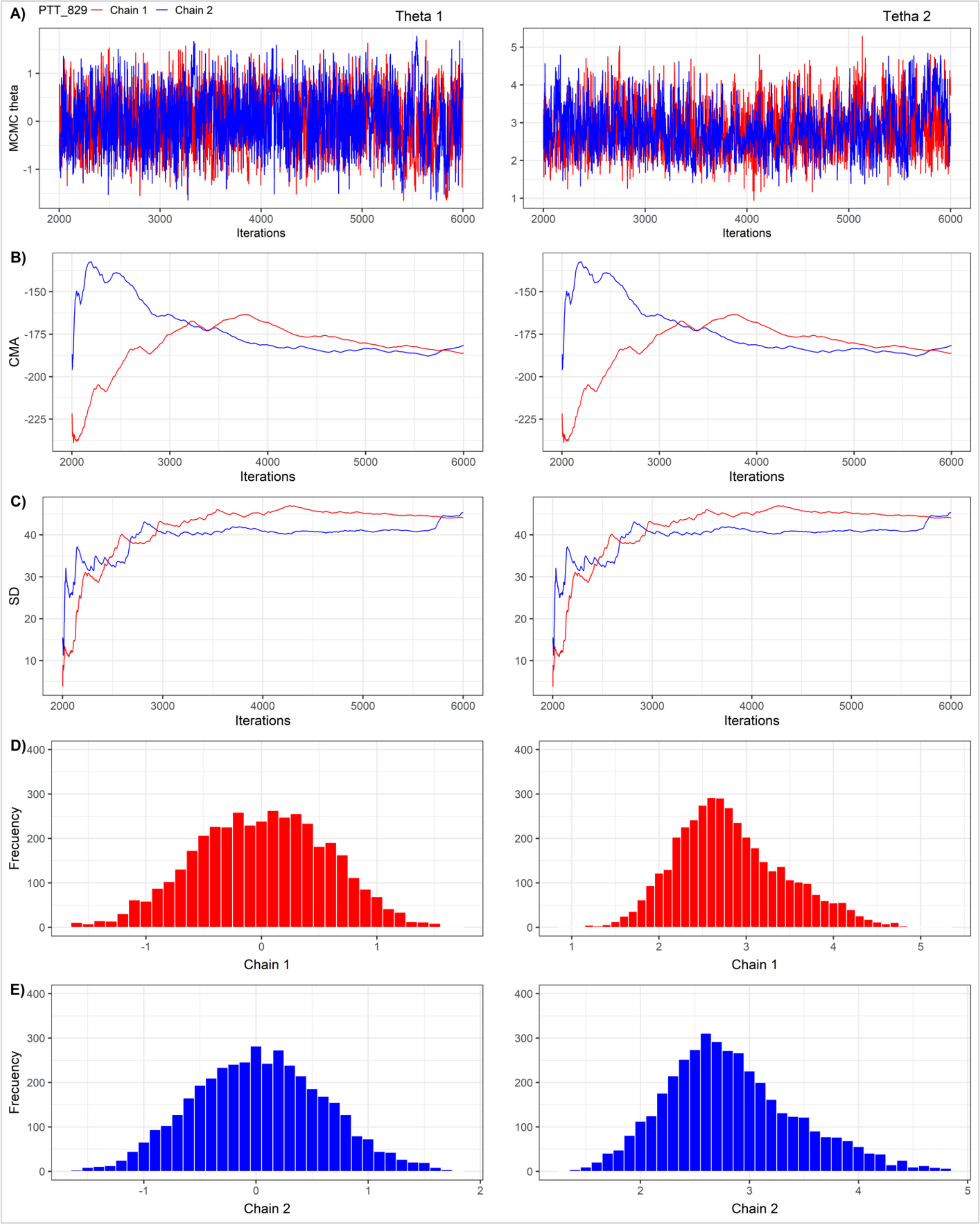
A)
PTT_829
Chain 1
Chain 2
Theta 1
Tetha 2
MCMC theta
Iterations
B)
CMA
Iterations
C)
SD
Iterations
D)
Frecuency
Chain 1
E)
Frecuency
Chain 2

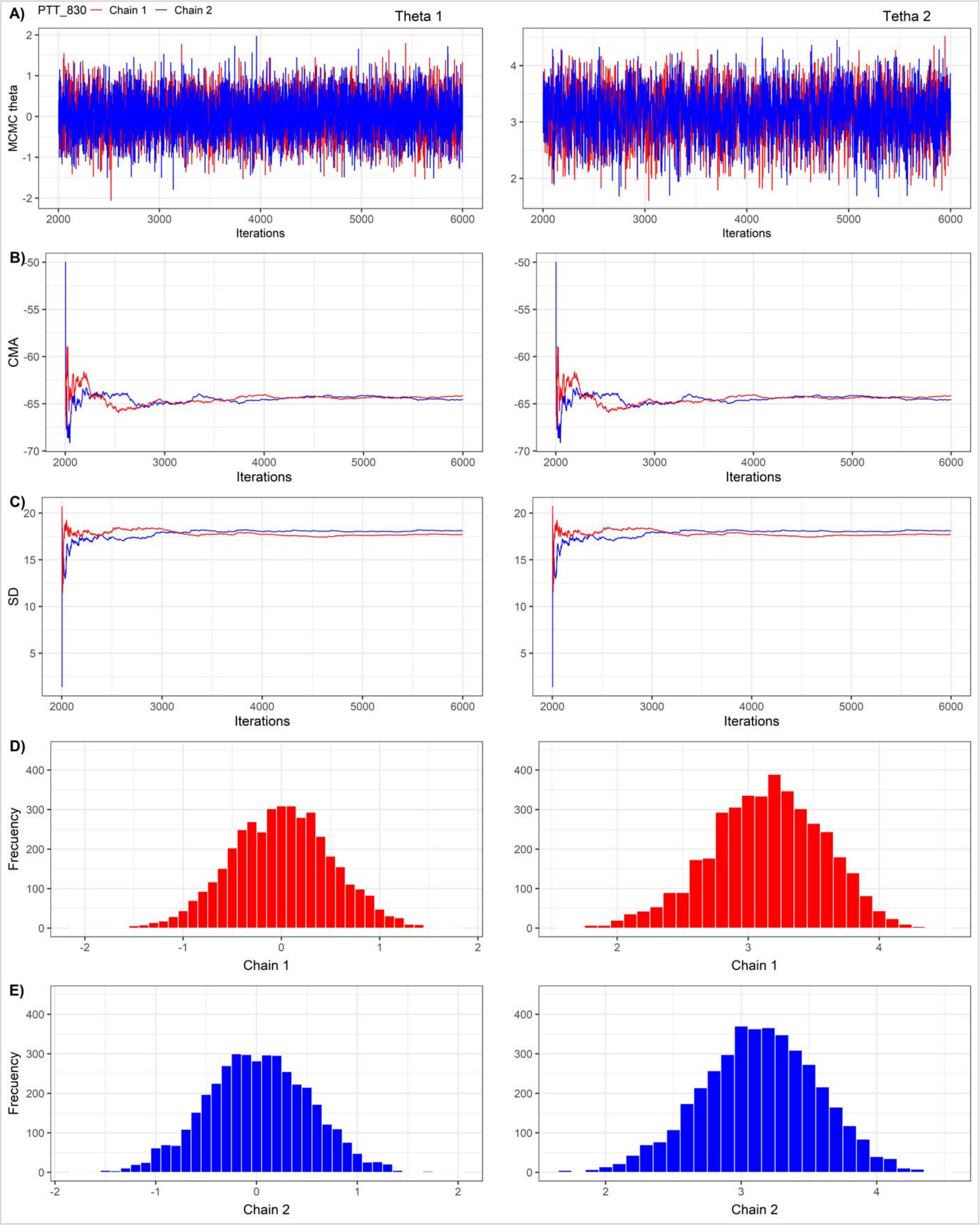
A)
PTT_830
Chain 1
Chain 2
Theta 1
Tetha 2
MCMC theta
Iterations
B)
CMA
Iterations
C)
SD
Iterations
D)
Frecuency
Chain 1
E)
Frecuency
Chain 2

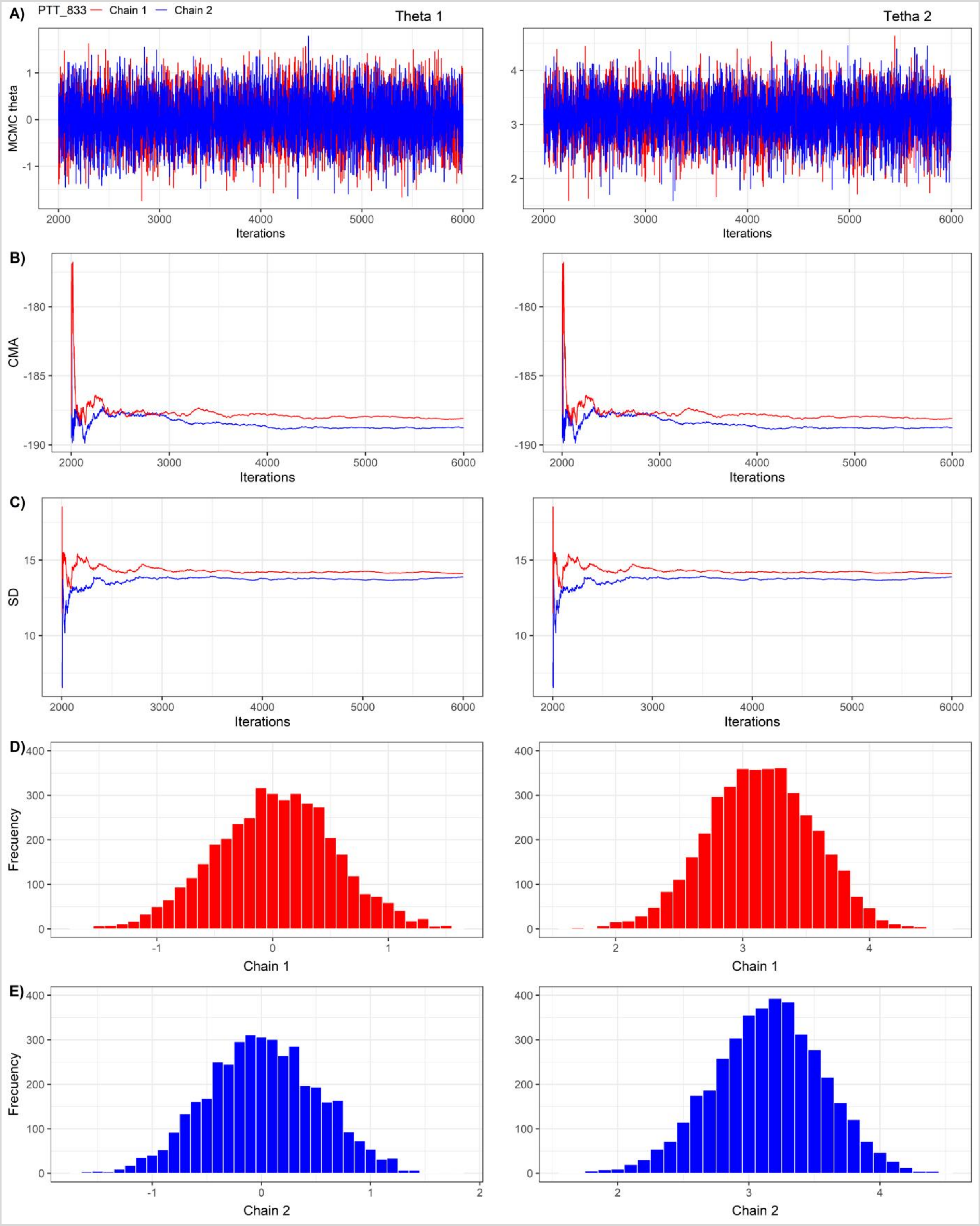
A)
PTT_833
Chain 1
Chain 2
Theta 1
Tetha 2
MCMC theta
Iterations
B)
CMA
Iterations
C)
SD
Iterations
D)
Frecuency
Chain 1
E)
Frecuency
Chain 2

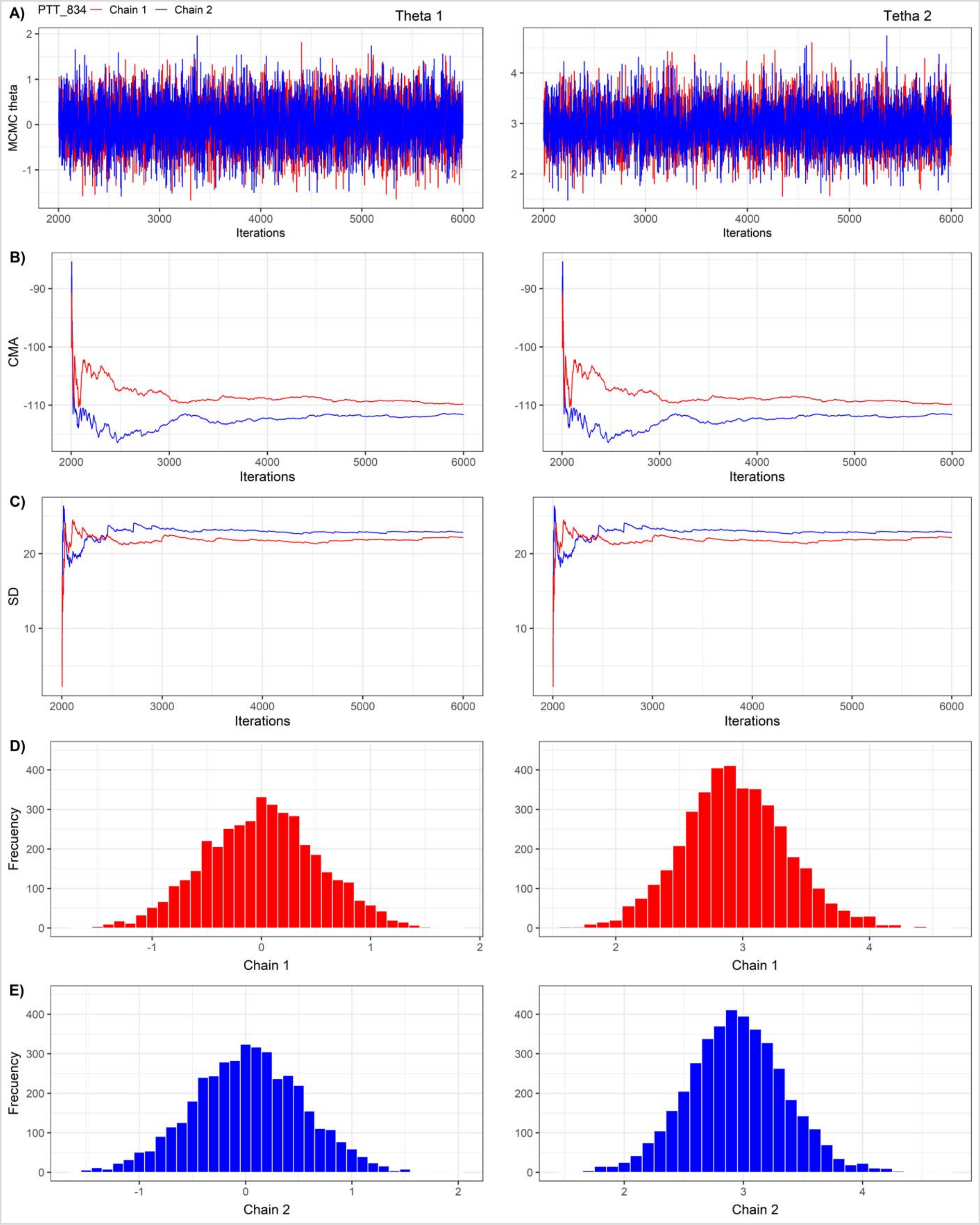
A)
PTT_834
Chain 1
Chain 2
Theta 1
Tetha 2
MCMC theta
Iterations
B)
CMA
Iterations
C)
SD
Iterations
D)
Frecuency
Chain 1
E)
Frecuency
Chain 2

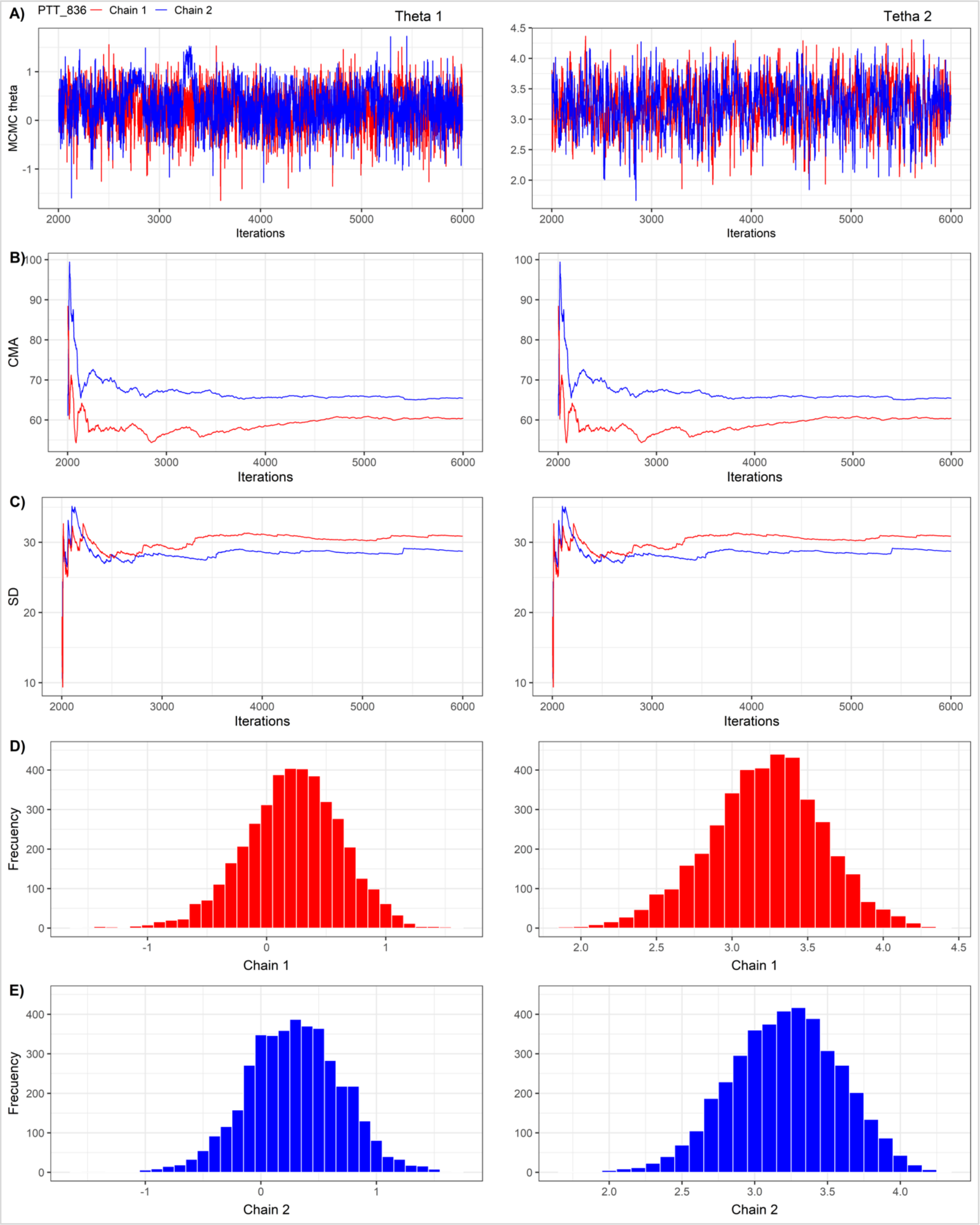
A)
PTT_836
Chain 1
Chain 2
Theta 1
Tetha 2
MCMC theta
Iterations
B)
CMA
Iterations
C)
SD
Iterations
D)
Frecuency
Chain 1
E)
Frecuency
Chain 2

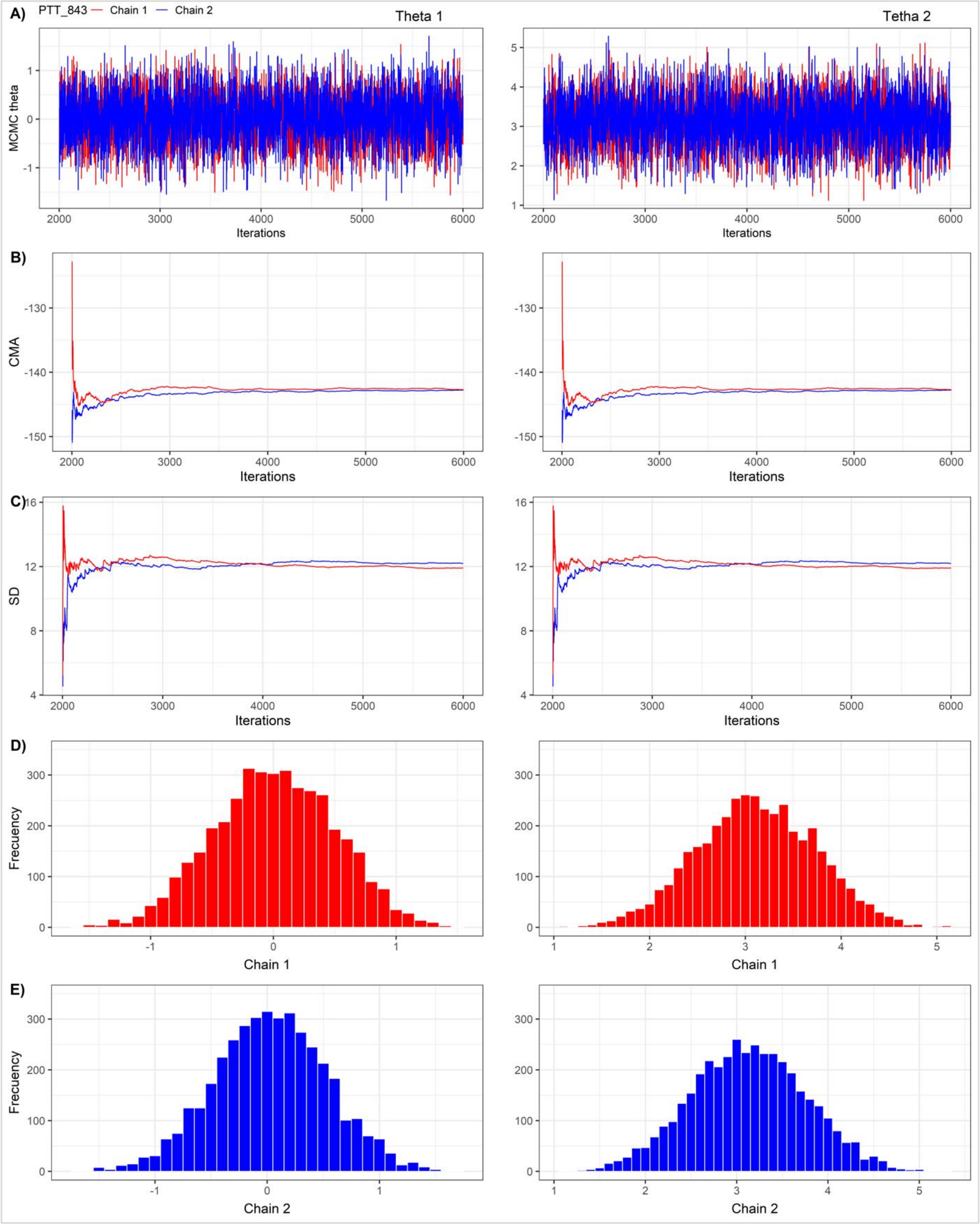

A)
PTT_843
Chain 1
Chain 2
Theta 1
Tetha 2
MCMC theta
Iterations
B)
CMA
Iterations
C)
SD
Iterations
D)
Frecuency
Chain 1
E)
Frecuency
Chain 2

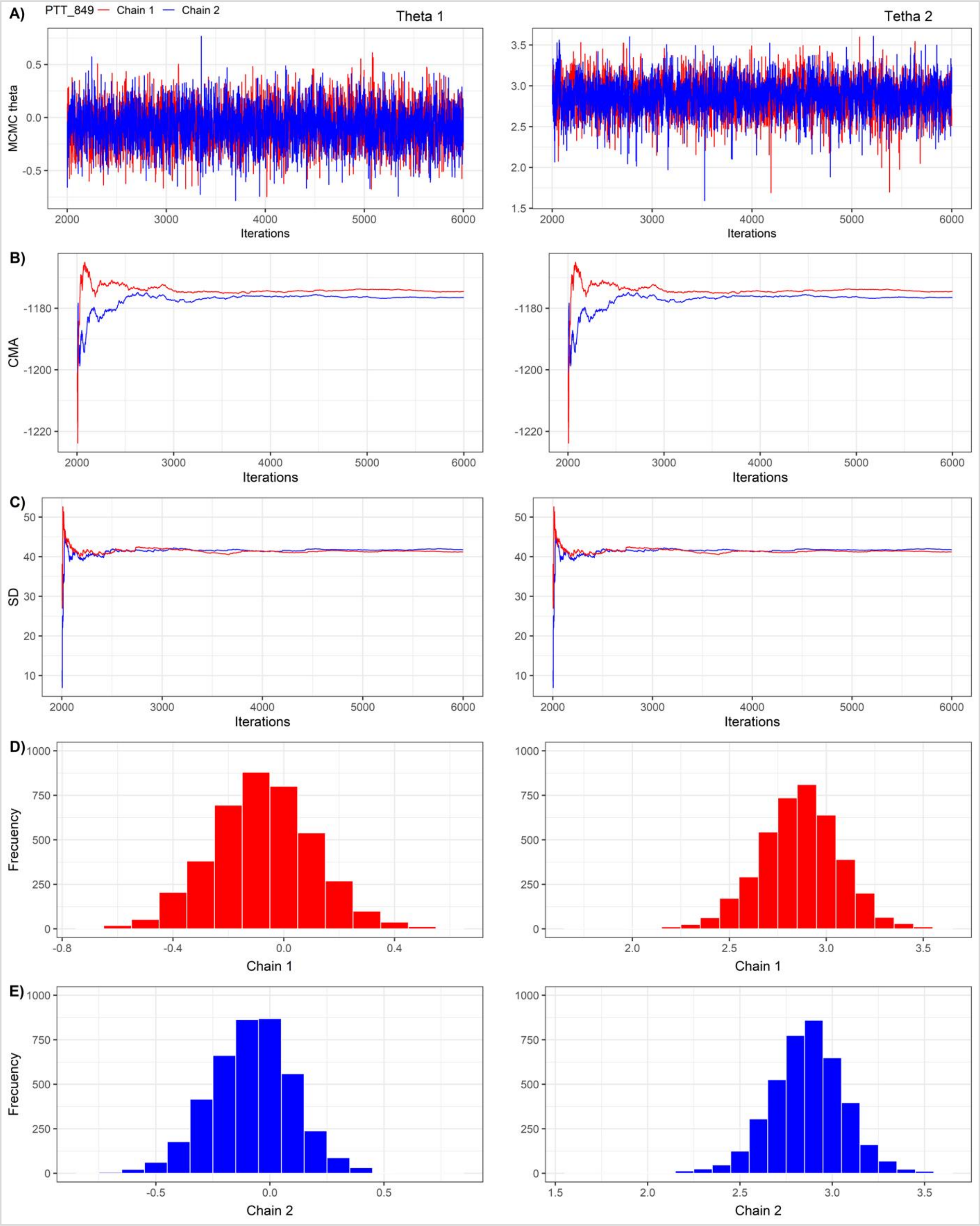

A)
PTT_849
Chain 1
Chain 2
Theta 1
Tetha 2
MCMC theta
Iterations
B)
CMA
Iterations
C)
SD
Iterations
D)
Frecuency
Chain 1
E)
Frecuency
Chain 2

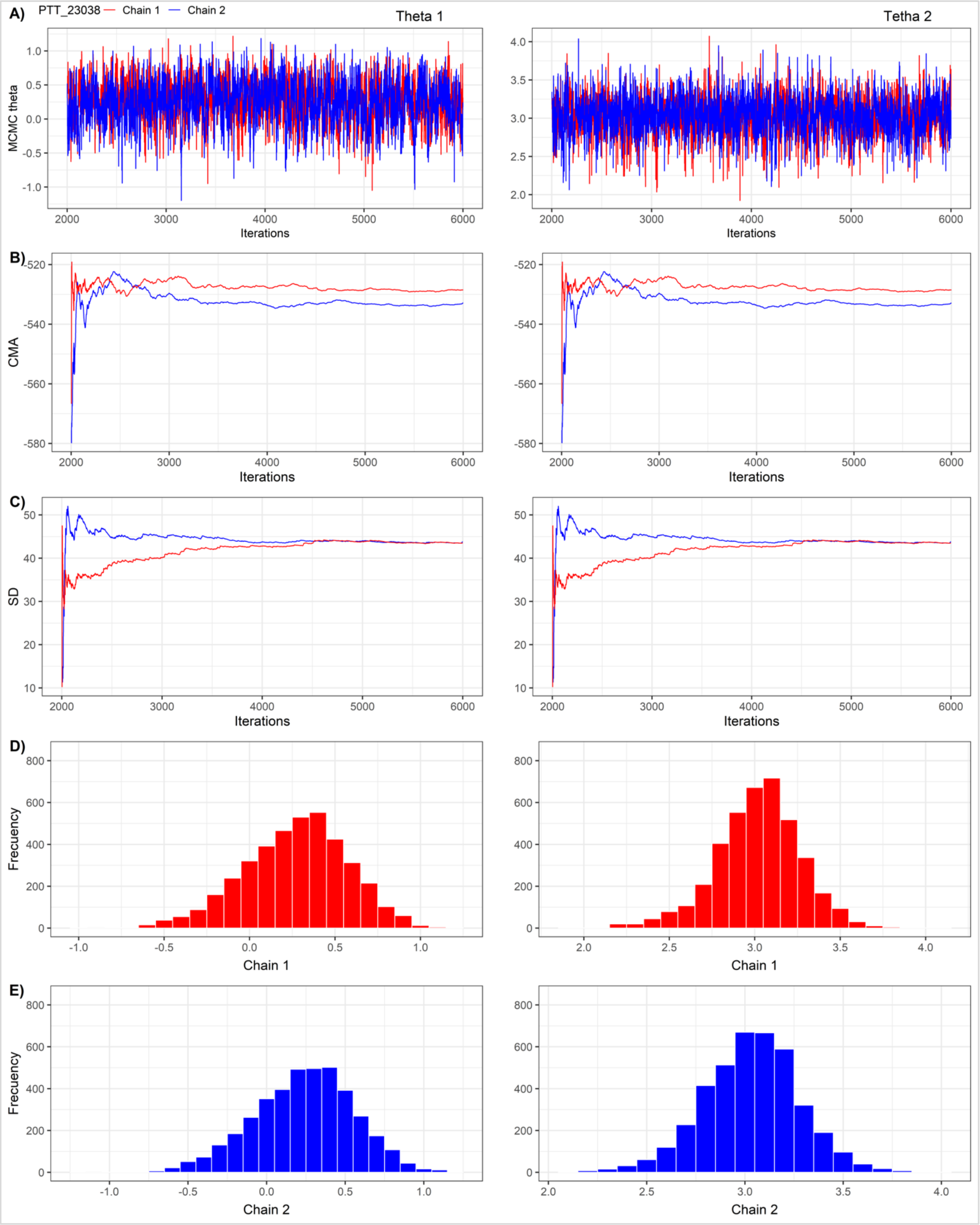
A)
PTT_23038
Chain 1
Chain 2
Theta 1
Tetha 2
MCMC theta
Iterations
B)
CMA
Iterations
C)
SD
Iterations
D)
Frecuency
Chain 1
E)
Frecuency
Chain 2

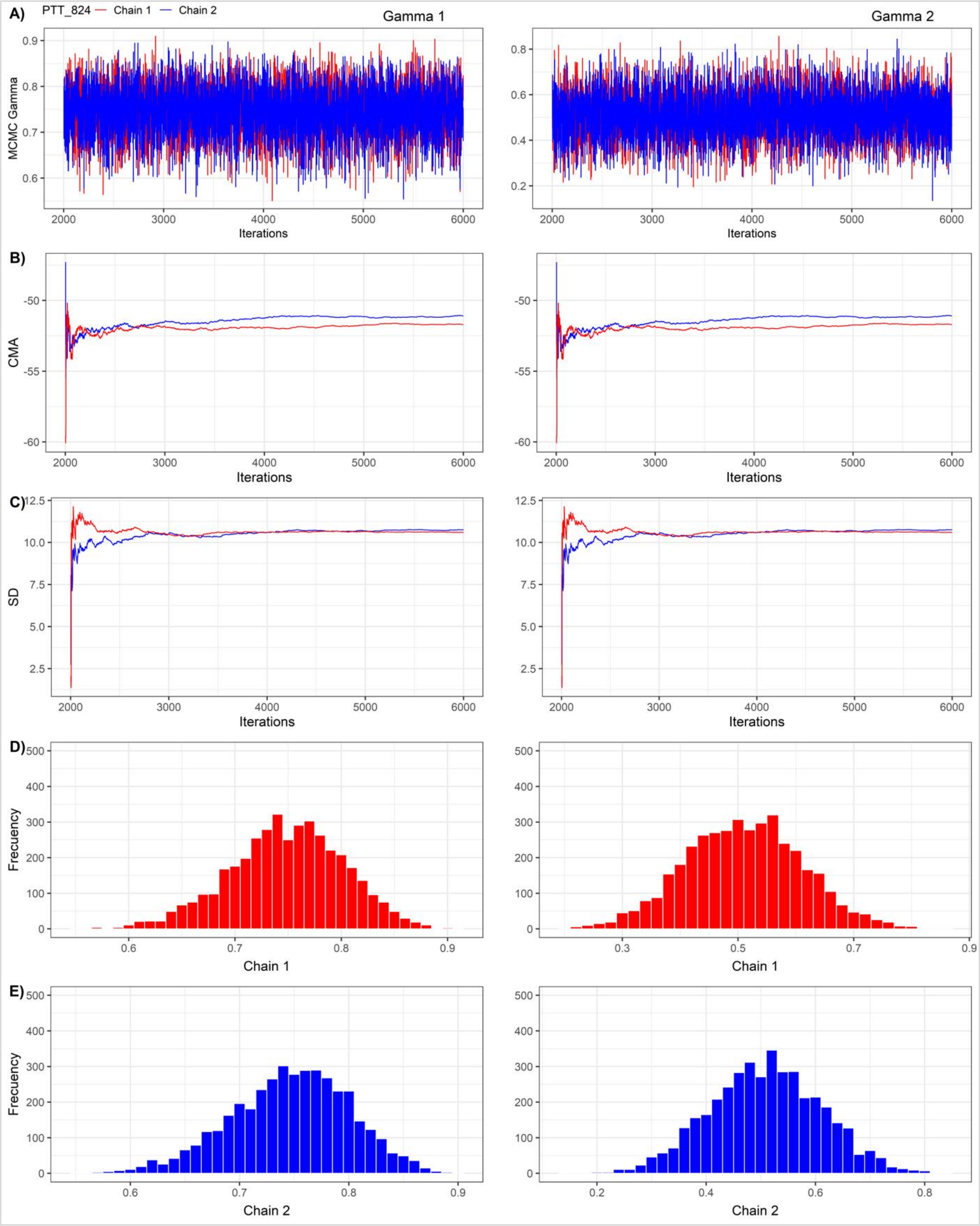
A)
PTT_824
Chain 1
Chain 2
Gamma 1
Gamma 2
MCMC Gamma
Iterations
B)
CMA
C)
SD
D)
Frecuency
Chain 1
E)
Chain 2

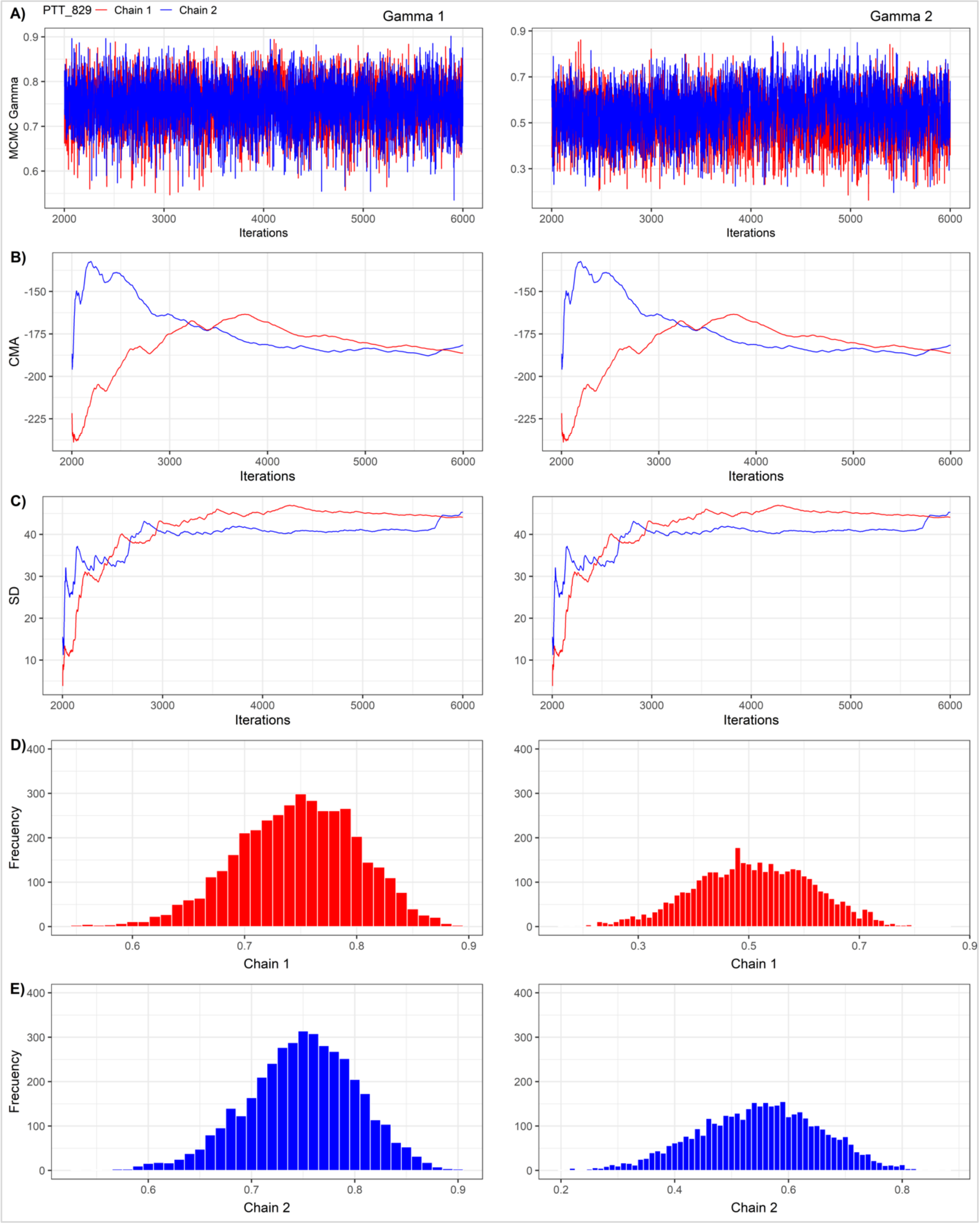

A)
PTT_829
Chain 1
Chain 2
Gamma 1
Gamma 2
MCMC Gamma
Iterations
B)
CMA
Iterations
C)
SD
Iterations
D)
Frecuency
Chain 1
E)
Frecuency
Chain 2

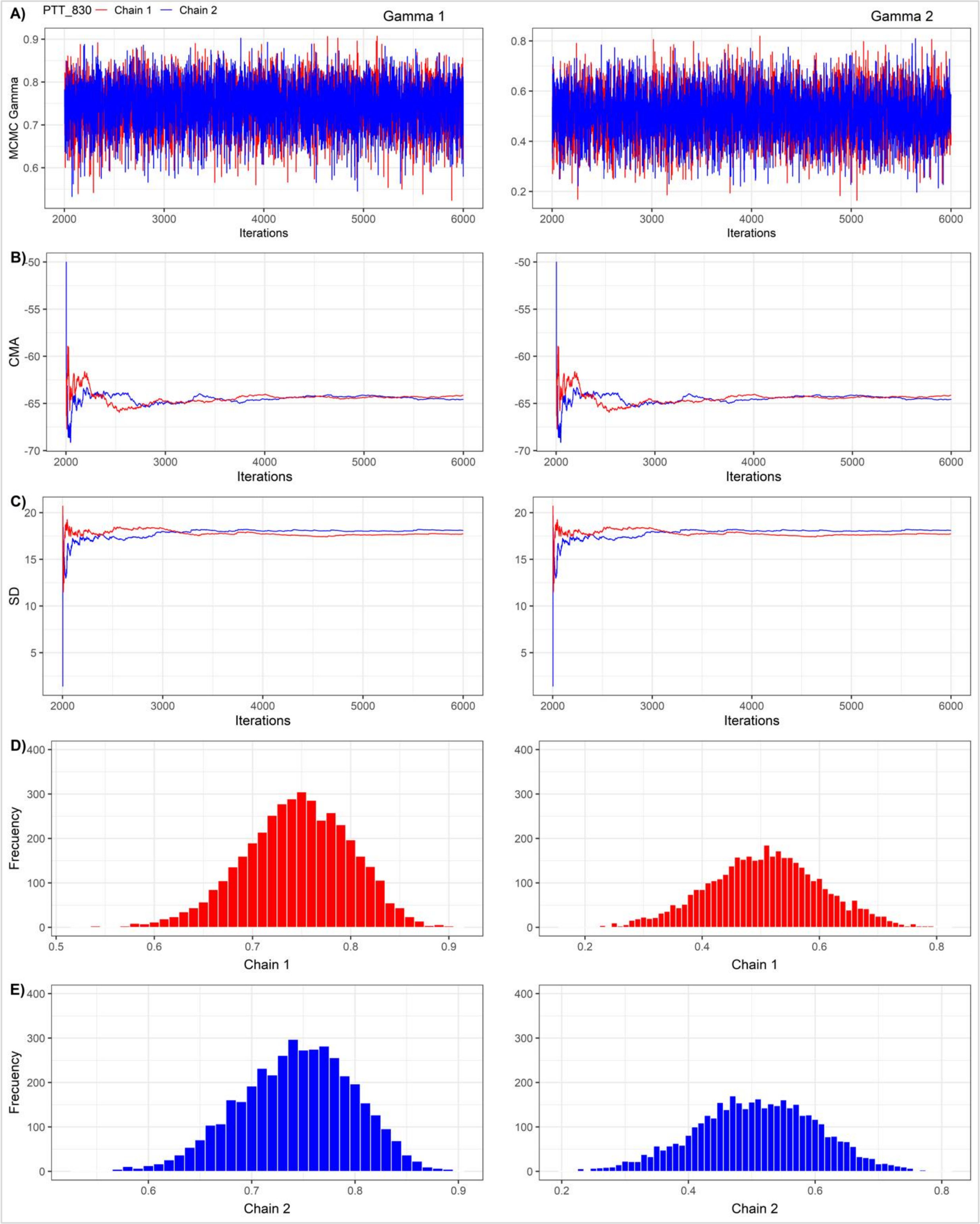

A)
PTT_830
Chain 1
Chain 2
Gamma 1
Gamma 2
MCMC Gamma
Iterations
B)
CMA
Iterations
C)
SD
Iterations
D)
Frecuency
Chain 1
E)
Frecuency
Chain 2

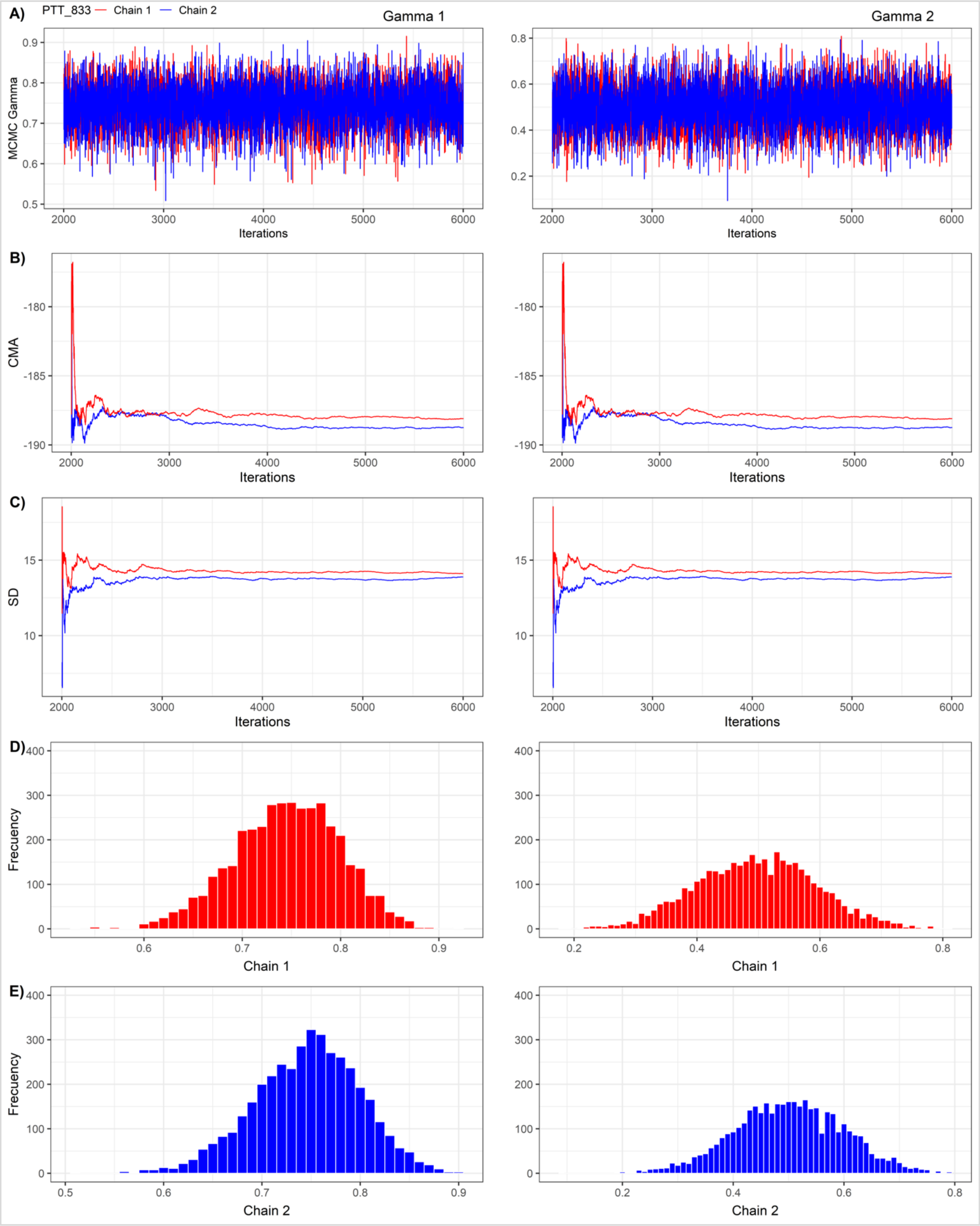
A)
PTT_833
Chain 1
Chain 2
Gamma 1
Gamma 2
MCMC Gamma
Iterations
B)
CMA
Iterations
C)
SD
Iterations
D)
Frecuency
Chain 1
E)
Frecuency
Chain 2

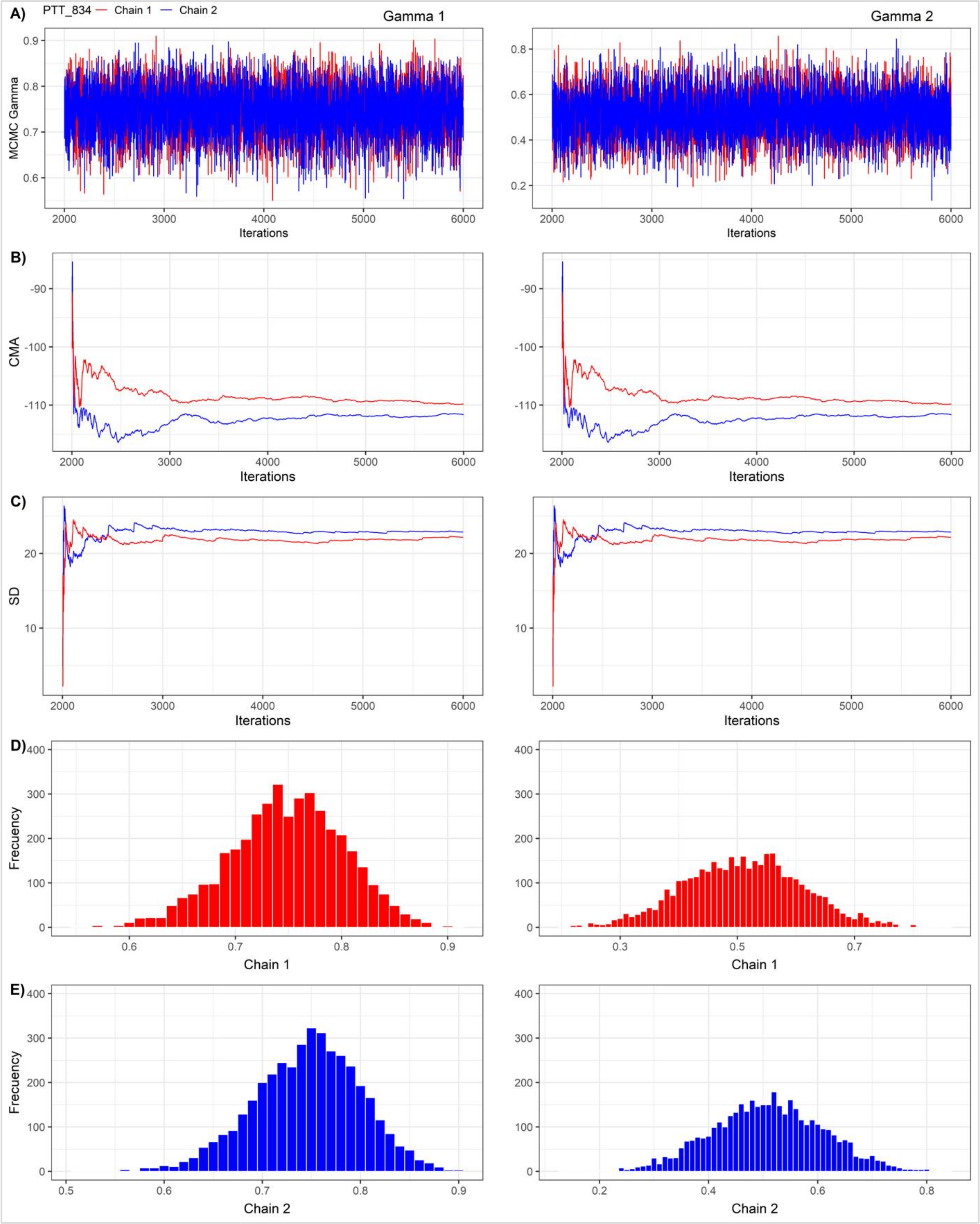

A)
PTT_834
Chain 1
Chain 2
Gamma 1
Gamma 2
MCMC Gamma
Iterations
B)
CMA
Iterations
C)
SD
Iterations
D)
Frecuency
Chain 1
E)
Frecuency
Chain 2

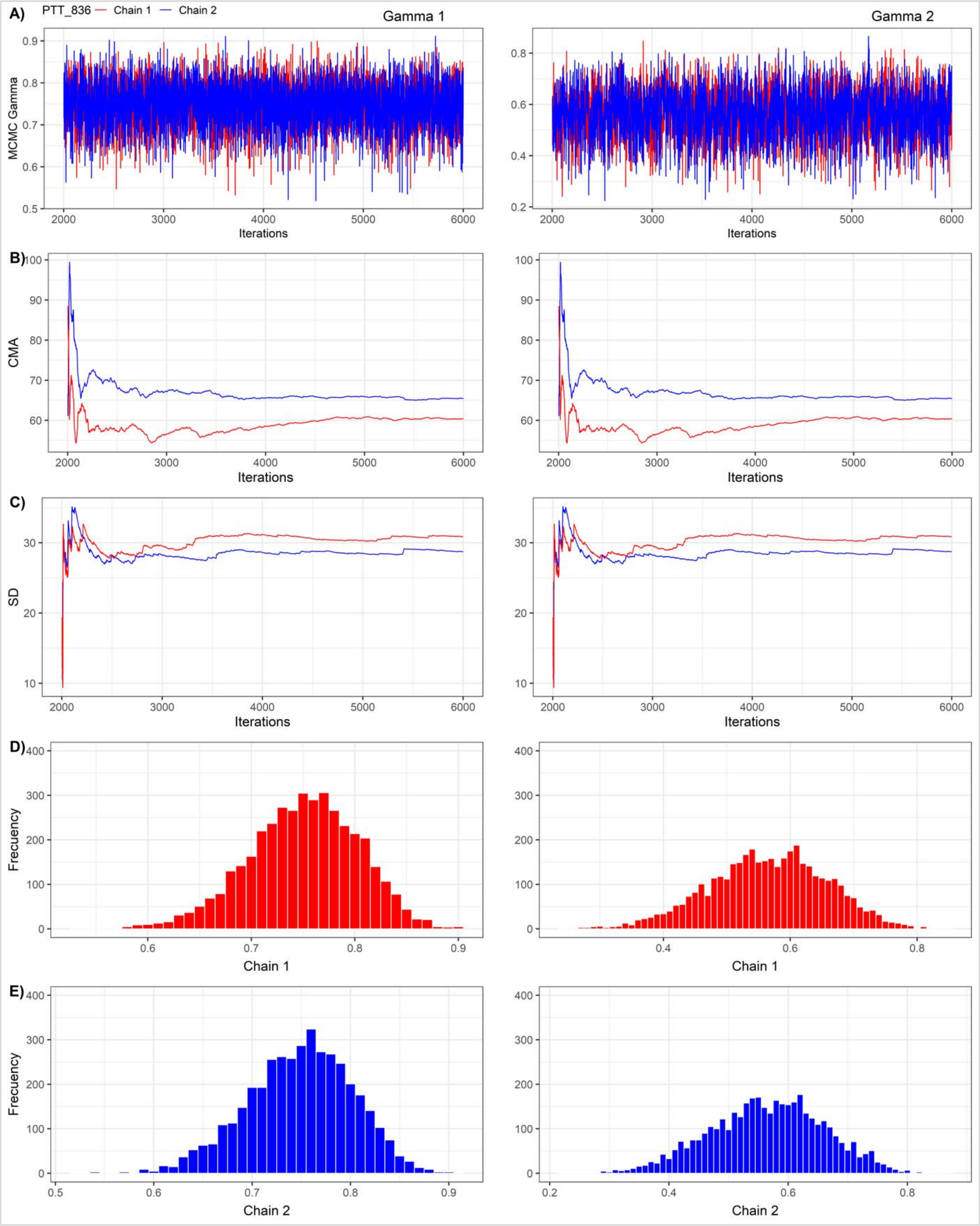
A)
PTT_836
Chain 1
Chain 2
Gamma 1
Gamma 2
MCMC Gamma
Iterations
B)
CMA
C)
SD
D)
Frecuency
Chain 1
E)
Chain 2

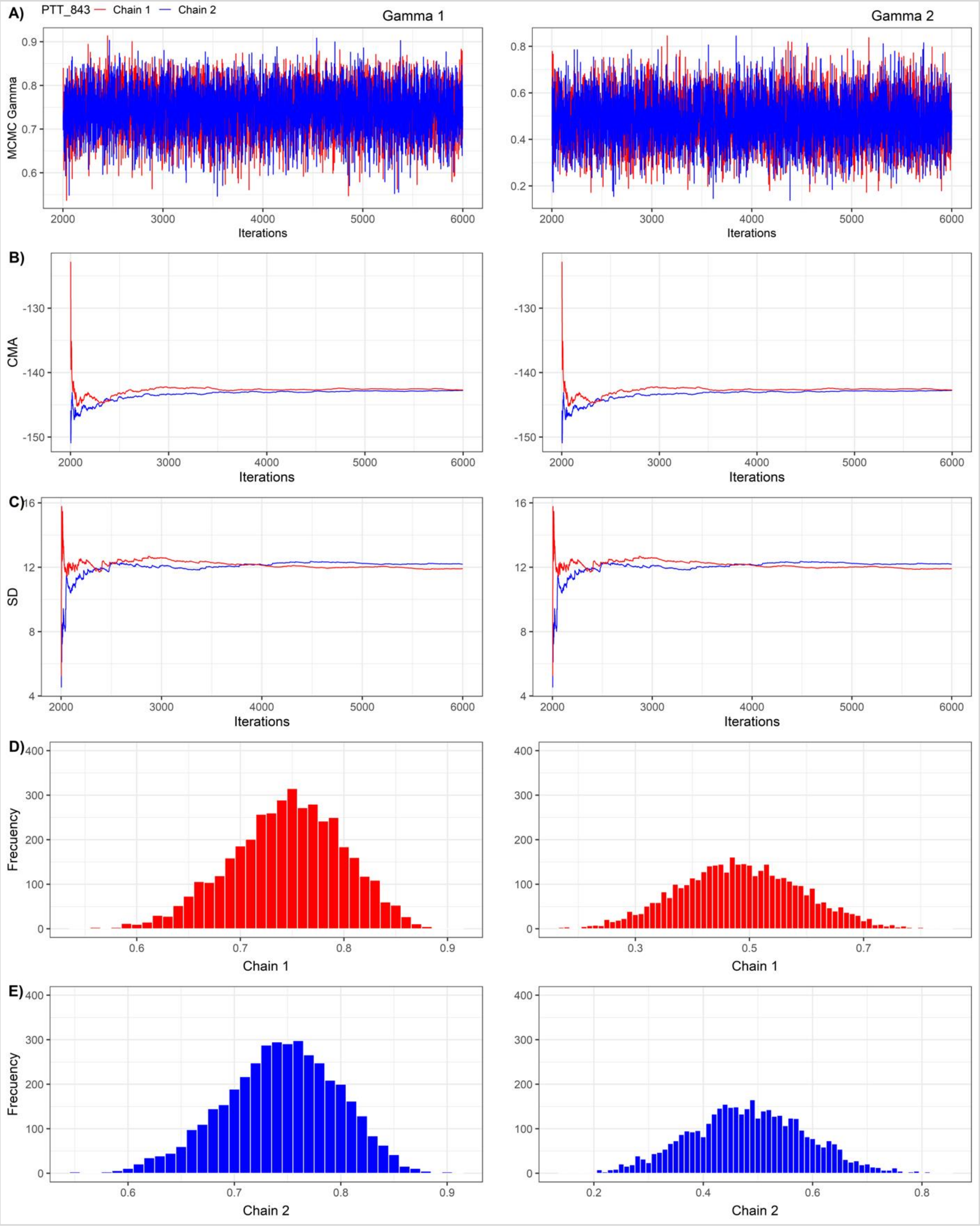
A)
PTT_843
Chain 1
Chain 2
Gamma 1
Gamma 2
MCMC Gamma
Iterations
B)
CMA
Iterations
C)
SD
Iterations
D)
Frecuency
Chain 1
E)
Frecuency
Chain 2

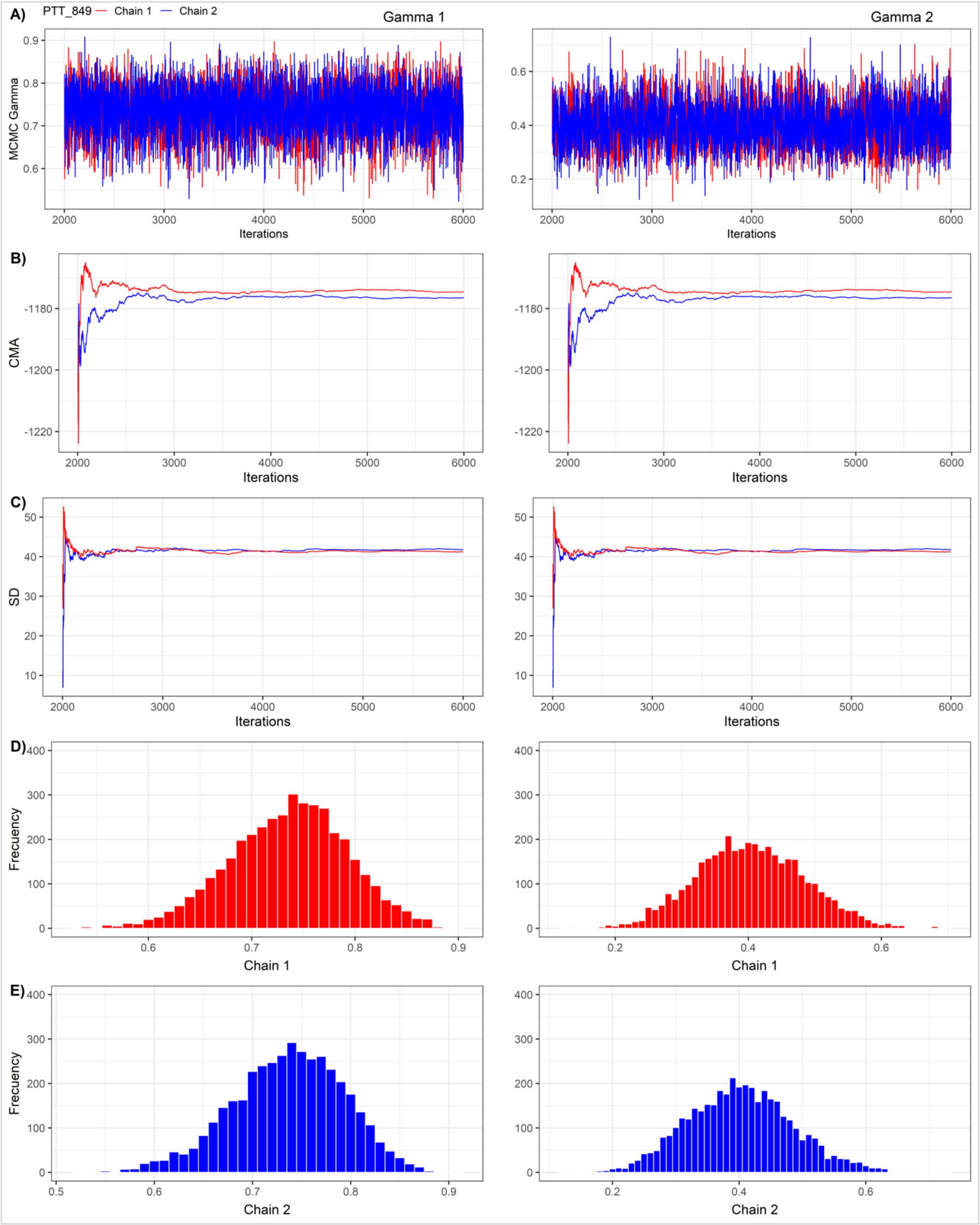
A)
PTT_849
Chain 1
Chain 2
Gamma 1
Gamma 2
MCMC Gamma
Iterations
B)
CMA
Iterations
C)
SD
Iterations
D)
Frecuency
Chain 1
E)
Frecuency
Chain 2

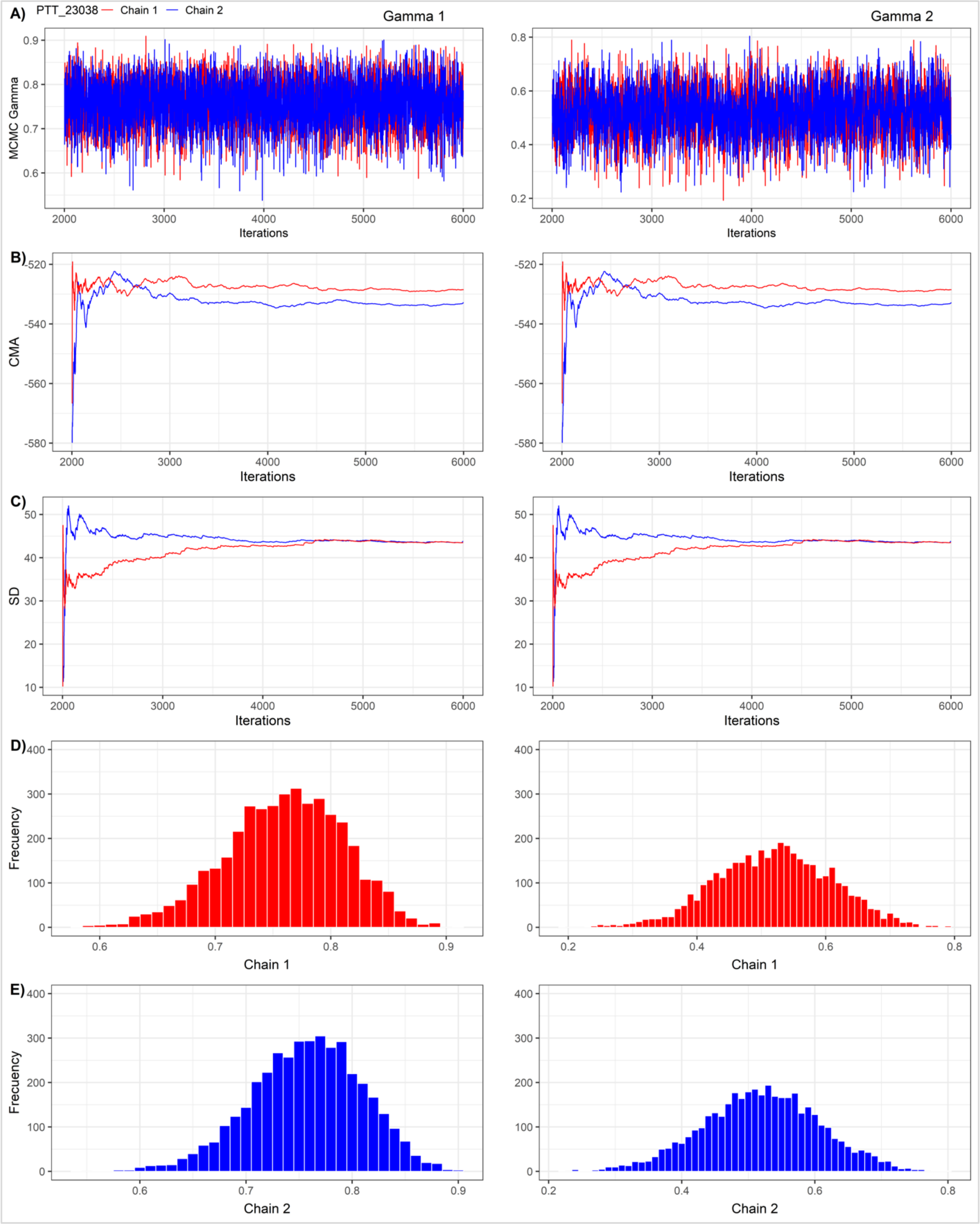
A)
PTT_23038
Chain 1
Chain 2
Gamma 1
Gamma 2
MCMC Gamma
Iterations
B)
CMA
Iterations
C)
SD
Iterations
D)
Frecuency
Chain 1
E)
Frecuency
Chain 2
