# Supplementary material for: Fin whale movements in the Gulf of California, Mexico, from satellite telemetry
Source: PLoS One. 2019 Jan 10;14(1):e0209324. doi: 10.1371/journal.pone.0209324 (PMC6328206; doi:10.1371/journal.pone.0209324)

Additional File 2

The SSM tracks for eight fin whales tagged in March 2001 in the southwestern Gulf of California, along the east coast of the Baja California Peninsula, Mexico. Blue lines represent the cool season, red lines represent the warm season, and the arrow the track's direction.

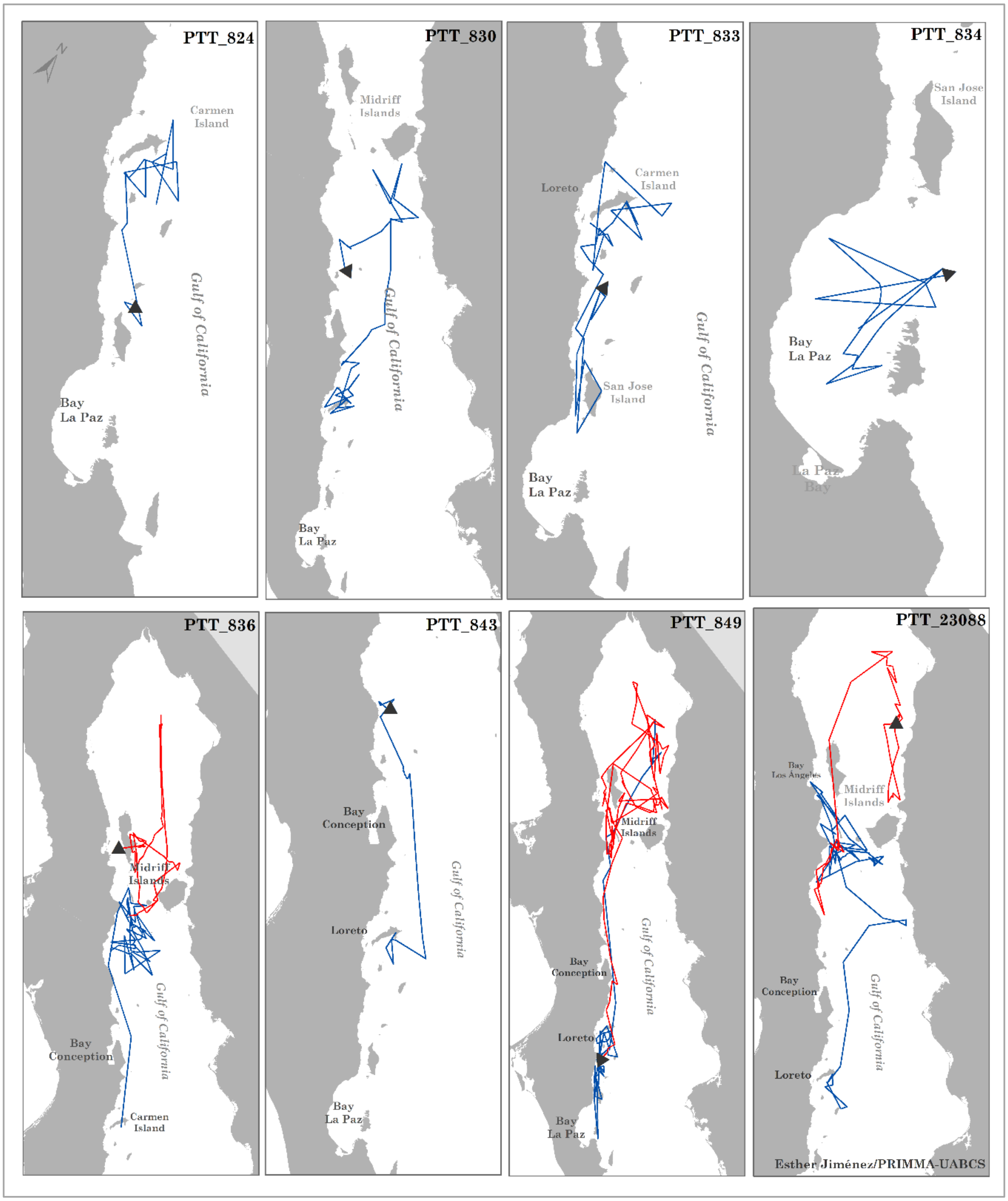

Probability density plots of the continuous behavioral mode values (bmode) predicted by the SSM for each location as the posterior means of the MCMC samples for each tag. Locations with bmode values greater than 1.75 were designated as in ARS mode and locations with values lower than 1.25 were designated as in transiting mode. Locations with behavioral state values in between these cutoffs were considered “uncertain”.

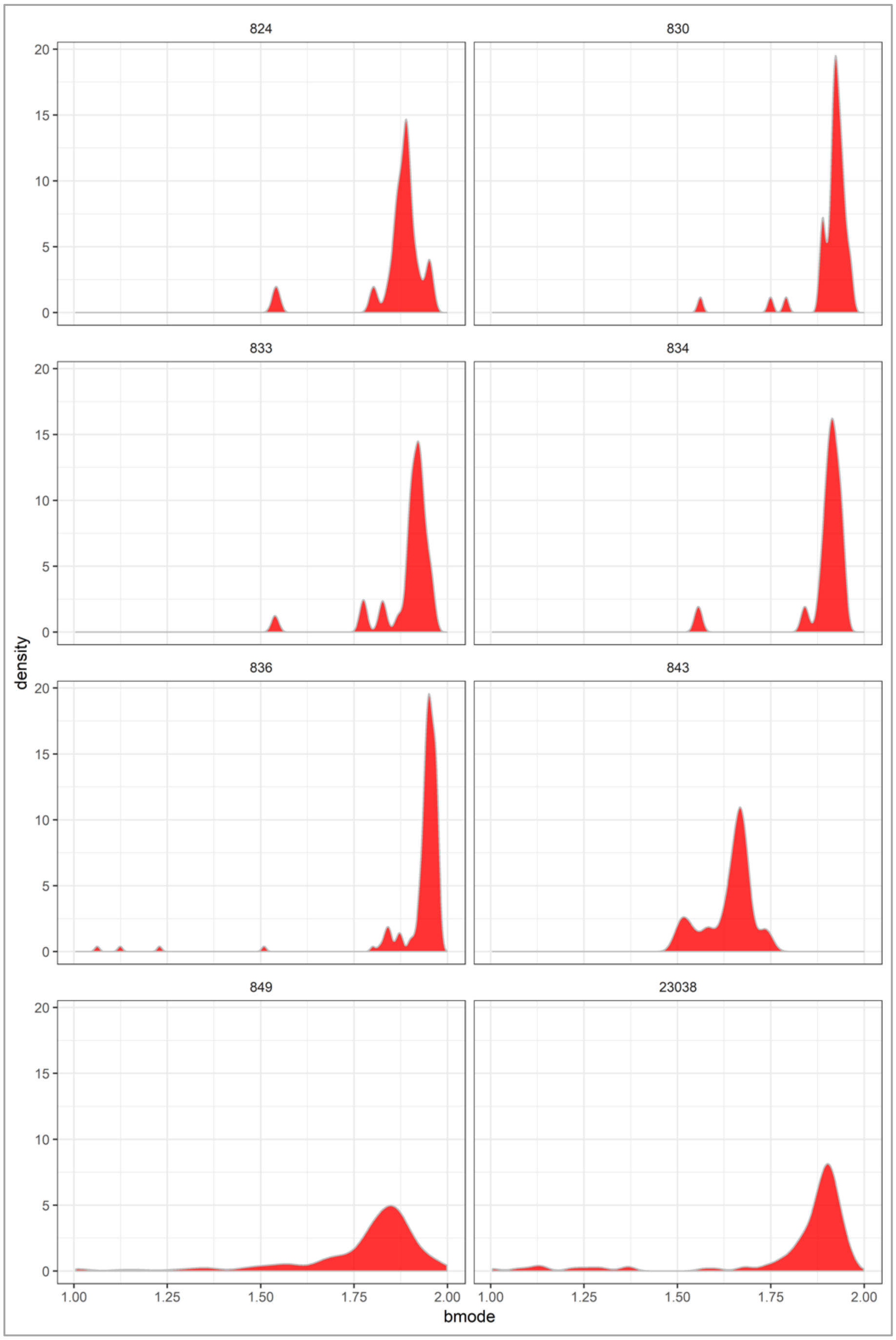

Supplement: S2 File — (PDF) [file pone.0209324.s002.pdf]
